# A new method for computing the projection median, its influence curve and techniques for the production of projected quantile plots

**DOI:** 10.1371/journal.pone.0229845

**Published:** 2020-05-07

**Authors:** Fan Chen, Guy Nason

**Affiliations:** 1 School of Mathematics, University of Bristol, Fry Building, Woodland Road, Bristol, England, United Kingdom; 2 Dept. Mathematics, Imperial College, London, England, United Kingdom; Huazhong University of Science and Technology, CHINA

## Abstract

This article introduces a new formulation of, and method of computation for, the projection median. Additionally, we explore its behaviour on a specific bivariate set up, providing the first theoretical result on form of the influence curve for the projection median, accompanied by numerical simulations. Via new simulations we comprehensively compare our performance with an established method for computing the projection median, as well as other existing multivariate medians. We focus on answering questions about accuracy and computational speed, whilst taking into account the underlying dimensionality. Such considerations are vitally important in situations where the data set is large, or where the operations have to be repeated many times and some well-known techniques are extremely computationally expensive. We briefly describe our associated R package that includes our new methods and novel functionality to produce animated multidimensional projection quantile plots, and also exhibit its use on some high-dimensional data examples.

## 1 Introduction: Overview of multivariate medians

The median is an estimator of location that is robust, i.e. not heavily influenced by outlying values, which are, loosely speaking, points that are far from the main body of the data. Let **x** = (*x*_1_, …, *x*_*k*_)^*T*^ be a mutually independent and identically distributed (i.i.d.) sample of length k∈N from a univariate distribution with distribution function *F*. The univariate population *median* functional *M*(*F*) is

$$\begin{array}{c} M\left(F\right)=\inf\{x:F(x)\geq 1/2\}=\sup\{x:F(x)\leq 1/2\}. \end{array}$$ (1)

There are several equivalent definitions of the univariate median that all yield same unique value of true median *μ* for a distribution *F* with a bounded and continuous density *f*(*μ*) at *μ*.

For multivariate data there is no natural ordering of the data to enable the choice of the middle observation in the same way as for one-dimensional data. However, several different multivariate median concepts have been developed that retain some characteristics of the univariate median. For example, an early extension of the multivariate median was suggested by Hayford [1], which is simply the component-wise median, also known as the vector of marginal medians. The spatial median, also known as the *L*_1_ median [2, 3], and Tukey’s median [4] are two other popular variants. Oja’s median [5] provides an alternative to the spatial median, but it is known to be more computationally expensive than other choices. These, and others, are reviewed in [6–8]. We briefly review some of them here next, not least as we use them later in our simulation study.

### 1.1 Component-wise median

Let **X** = (**x**_1_, …, **x**_*k*_)^*T*^ be an *n*-dimensional i.i.d. sample with distribution function $F:R^{n}\to R$. We assume that the *n* marginal distributions have bounded densities *f*_1_(*μ*_1_), …, *f*_*n*_(*μ*_*n*_) at the uniquely defined marginal medians *μ* = (*μ*_1_, …, *μ*_*n*_). The component-wise median, also known as the marginal sample median, $M_{C}\left(X\right)\in R^{n}$ minimises

$$\begin{array}{c} k^{-1}\sum_{i=1}^{k}\{(|x_{i1}-m_{1}\left|+\cdots +\right|x_{in}-m_{n}\left|\right)-\left(\right|x_{i1}\left|+\cdots +\right|x_{in}|)\}, \end{array}$$ (2)

the sum of component-wise distances over $m\in R^{n}$, where **m** = (*m*_1_, …, *m*_*n*_). The corresponding population functional, *M*_*C*_(*F*), for the vector of population medians minimises

$$\begin{array}{c} E\{(|x_{1}-m_{1}\left|+\cdots +\right|x_{n}-m_{n}\left|\right)-\left(\right|x_{1}\left|+\cdots +\right|x_{n}|)\}. \end{array}$$ (3)

### 1.2 Spatial median

The spatial median *M*_*S*_(**X**), also known as the *L*_1_ median, minimises

$$\begin{array}{c} k^{-1}\sum_{i=1}^{k}\{\left|\right|{\text{x}}_{i}-\text{m}||-\left|\right|{\text{x}}_{i}\left|\right|\}, \end{array}$$ (4)

over $\text{m}\in R^{n}$, where ${\left||\text{m}|\right|}^{2}=\sum_{i=1}^{n}m_{i}^{2}$ is the (squared) Euclidean norm. The corresponding functional spatial median, *M*_*S*_(*F*), minimises

$$\begin{array}{c} E_{F}\{||\text{x}-\text{m}||-||\text{x}||\}. \end{array}$$ (5)

### 1.3 Oja’s median

Let **X** = (**x**_1_, …, **x**_*k*_)^*T*^ be an i.i.d. sample in $R^{n}$ with distribution function $F:R^{n}\to R$. The volume of the *n*-variate simplex determined by the *n* + 1 vertices (**m**_1_, …, **m**_*n*+1_) is

$$\begin{array}{c} V\left({\text{m}}_{1},\ldots ,{\text{m}}_{n+1}\right)=\frac{1}{p!}\;|det(\begin{array}{ccc} 1 & \cdots & 1\\ {\text{m}}_{1} & \cdots & {\text{m}}_{n+1} \end{array})|. \end{array}$$ (6)

The Oja median, *M*_*O*_(**X**), minimises

(kn)-1∑i1<⋯<inV(xi1,…,xin,m), (7)

over $\text{m}\in R^{n}$. The corresponding functional *M*_*O*_(*F*) minimises

$$\begin{array}{c} E_{F}\{V({\text{x}}_{i_{1}},\ldots ,{\text{x}}_{i_{n}},\text{m})\}. \end{array}$$ (8)

### 1.4 Tukey’s median

Let **X** = (**x**_1_, …, **x**_*k*_)^*T*^ be an i.i.d. sample of size *k* in $R^{n}$ with distribution function $F:R^{n}\to R$. Let H be the class of all closed half spaces in $R^{n}$. For each H∈H, define the empirical distribution

$$\begin{array}{c} \hat{F}\left(H\right)=n^{-1}\sum_{i=1}^{k}I\left({\text{x}}_{i}\in H\right), \end{array}$$ (9)

where I is the usual indicator function. Then, define the *depth*, *D*(***μ***), of a point $\mu \in R^{n}$ within the dataset, to be the infinum of $\hat{F}\left(H\right)$, that is taken over all closed half spaces *H* for which ***μ*** ∈ *H*. Tukey’s median is defined as the set of points ***μ*** of maximal depth.

## 2 The projection median

This section introduces our new method for computing the projection median, yamm. We prove that yamm is equivalent to the projection median, as defined by Durocher and Kirkpatrick [9] in $R^{2}$ and then generalised to higher dimensions by Basu *et al*. [10]. We also explore, theoretically and numerically, the statistical behaviour of yamm using a mixture of two bivariate normal distributions.

### 2.1 Review of the projection median

#### 2.1.1 Projection median in $R^{2}$

Let **X** be a multiset of points in $R^{2}$ and *θ* ∈ [0, 2*π*) be an angle. Let **X**_*θ*_ denote the multiset defined by the projection of **X** onto the unit vector *u*_*θ*_ = (cos *θ*, sin *θ*), so

$$\begin{array}{c} {\text{X}}_{\theta }=\{u_{\theta }\left\langle \text{x},u_{\theta }\right\rangle \;\;|\;\;\text{x}\in \text{X}\}, \end{array}$$ (10)

where 〈⋅〉 denotes the usual inner product.

The projection median of a non-empty finite set **X** with points in $R^{2}$ is

$$\begin{array}{c} M_{P}\left(\text{X}\right)=\pi^{-1}\int_{0}^{2\pi }\text{med}\left({\text{X}}_{\theta }\right)\;d\theta , \end{array}$$ (11)

where $\text{med}\left({\text{X}}_{\theta }\right)\in R^{2}$ is the median of the projection of **X** onto the line through the origin, parallel to *u*_*θ*_.

#### 2.1.2 Generalisation of the projection median

Given a fixed positive integer, *n* ≥ 2, and a finite set of points **X** in $R^{n}$, the *n*-dimensional projection median of **X** is

$$\begin{array}{c} M_{P}\left(\text{X}\right)=n\frac{\int_{{\text{X}}^{n-1}}\text{med}\left({\text{X}}_{a}\right)da}{\int_{{\text{X}}^{n-1}}da}=n\;\int_{{\text{X}}^{n-1}}\text{med}\left({\text{X}}_{a}\right)df\left(a\right), \end{array}$$ (12)

where ${\text{X}}^{n-1}=\{\text{x}\in R^{n}:||\text{x}||=1\}$ is the unit *n*-dimensional hypersphere, med(**X**_**a**)_ is the median of the projection of **X** onto the line through the origin parallel to **a**, and *f* is the normalised uniform measure over **X**^*n*−1^. Hence, for a point **x** = (*x*_1_, *x*_2_, …, *x*_*n*_)∈**X**^*n*−1^, the *n*-dimensional spherical coordinates are given by

$$\begin{array}{cc} x_{1} & =\cos\theta_{1}\\ x_{2} & =\sin\theta_{1}\cos\theta_{2}\\ x_{3} & =\sin\theta_{1}\sin\theta_{2}\cos\phi_{3}\\ & \;\;\cdots \\ x_{n-1} & =\sin\theta_{1}\cdots \sin\theta_{n-2}\cos\theta_{n-1}\\ x_{n} & =\sin\theta_{1}\cdots \sin\theta_{n-2}\sin\theta_{n-1}, \end{array}$$ (13)

where each angle *θ*_1_, *θ*_2_, …, *θ*_*n*−2_ has a range of *π* and *θ*_*n*−1_ has range of 2*π*. Also, the normalised uniform measure *f* over **X**^*n*−1^ is given by

$$\begin{array}{c} df=\frac{d_{{\text{X}}^{n-1}}V}{\int_{0}^{\pi }\int_{0}^{\pi }\cdots \int_{0}^{2\pi }d_{{\text{X}}^{n-1}}V}, \end{array}$$ (14)

where ${\text{d}}_{{\text{X}}^{n-1}}V=\sin^{n-2}\theta_{1}\;\sin^{n-3}\theta_{2}\ldots \sin\theta_{n-2}\;d\theta_{1}d\theta_{2}\ldots d\theta_{n-1}$ is the volume element of the (*n* − 1)-sphere.

Basu *et al*. [10] proved that the projection median has a breakdown point of 1/2 for all *n* ≥ 2.

### 2.2 Yet another multivariate median (Yamm)

Let $\text{X}={\left({\text{x}}_{1},\ldots ,{\text{x}}_{k}\right)}^{T}\in R^{k\times n}$ be a random sample of size k∈N, ${\text{x}}_{i}\in R^{n}$. Let **a** be a *n* × 1 projection vector of unit length, 1_*k*_ be the *k* × 1 vector of ones and ***μ*** a shift vector of length *n*. Let **y** be the projection of **X** onto **a** after **X** has been shifted by ***μ***:

$$\begin{array}{c} \text{y}=(\text{X}-1_{k}\;\mu^{T})\;\text{a}, \end{array}$$ (15)

where $\text{y}\in R^{k}$. The univariate median *m* of the projected points **y** is

$$\begin{array}{c} m_{\text{X}}\left(\mu ,\text{a}\right)=m\left(\text{y}\right). \end{array}$$ (16)

Now define the integral

$$\begin{array}{c} M_{\text{X},m}\left(\mu \right)=\int_{\left\{\text{a}:{\text{a}}^{T}\text{a}=1\right\}}m_{\text{X}}{\left(\mu ,\text{a}\right)}^{2}d\text{a}. \end{array}$$ (17)

The yamm estimator of location for **X** is

$$\begin{array}{c} \hat{\mu }=\text{yamm}\left(\text{X}\right)={\text{argmin}}_{\mu }M_{\text{X},m}\left(\mu \right). \end{array}$$ (18)

Eqs (17) and (18) illustrate the rationale behind yamm. Intuitively, if the shift vector ***μ*** is far away from the true ‘middle’ of the dataset, then the magnitude of *m*_**X**_(***μ***, **a**), as well as the integral *M*_**X**, *m*_(***μ***), will be large. By contrast, a smaller *m*_**X**_(***μ***, **a**) can be obtained when the ***μ*** is moving closer to the true ‘middle’ of the data set.

Instead of computing the squared value of *m*_**X**_(***μ***, **a**) for the integral, we also considered the absolute value as an alternative. However, this leads to similar numerical results.

**Fig 1:**
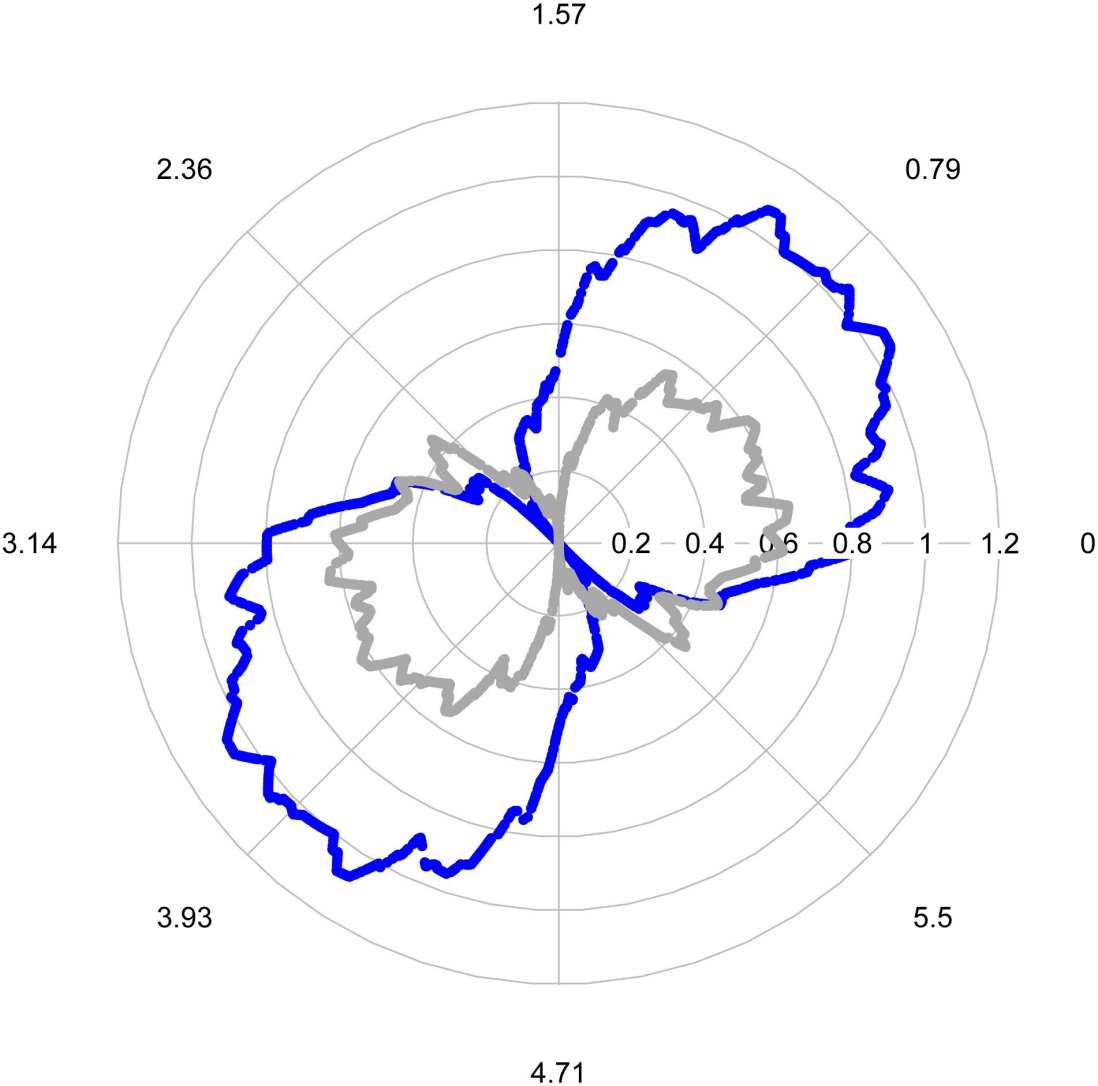
Polar plot (in radians) of the magnitude of *m*_X_(*μ*, a). *Grey line*: ***μ*** = (2.2, 8) and *Blue line*: ***μ*** = (2, 7.5).

*Example*. We now generate two polar plots of the absolute value of *m*_**X**_(***μ***, **a**), when ***μ*** is both close to, and far away, from the true median, respectively. A random two-dimensional dataset with *k* = 100 points was generated, whose Tukey’s median computed as (2.78, 8.16). Here, the Tukey median is to be interpreted as a ‘sensible’ middle of the data set. The shift vector ***μ*** is set to be (2.2, 8) and (2, 7.5) respectively, and for each plot, two thousand random projections were used to calculate the univariate median *m*_**X**_(***μ***, **a**), using methods to be explained in Section 2.4. Fig 1 shows that when ***μ*** is near the Tukey’s median, the magnitude of each *m*_**X**_(***μ***, **a**) is less than 0.65, while a larger value, ranging from 0 to 1.2, is shown in the figure when ***μ*** is far away from the median. Overall, when integrated the quantity involving the ***μ*** is closer to the Tukey median it gives a smaller result.

The projection median and yamm definitions seem similar, as both project the multiset onto the line passing through the origin, and then take the median. However, the projection median integrates med(**X**_**a**_) directly over the unit hypersphere in $R^{n}$, whereas yamm minimises the objective function $M_{\text{X},m}\left(\mu \right)\in R$ over the shift vector ***μ***. Despite these differences, the following theorem shows that the projection median and yamm are identical.

**Theorem**. *For any finite multiset* $\text{X}\subseteq R^{n}$ *with n* ≥ 2, *yamm is equivalent to the projection median*.

For the proof of the theorem, see S1 Appendix.

### 2.3 Yamm behaviour on a bivariate normal mixture

To gain insight about the theoretical behaviour of yamm we study the case of yamm applied to a mixture of two bivariate normals, where one is thought of as the bulk and the other as the outlier of the distribution. Such a setup enables us to evaluate the robustness of yamm. We numerically and theoretically assess the influence curve when moving the outlier far from the bulk.

#### 2.3.1 Bivariate mixture setup

Let ${\text{X}}_{1}∼N_{2}(\nu_{1},\Sigma_{1})$ and ${\text{X}}_{2}∼N_{2}(\nu_{2},\Sigma_{2})$ be independent bivariate normal random variables, where **X**_1_ = (*X*_11_, *X*_12_)^*T*^, **X**_2_ = (*X*_21_, *X*_22_)^*T*^ with mean vector ***ν***_1_ = (*ν*_11_, *ν*_12_)^*T*^ and ***ν***_2_ = (*ν*_21_, *ν*_22_)^*T*^. Let **R**(*θ*) be a rotation matrix with angle *θ* given by

$$\begin{array}{c} R\left(\theta \right)=(\begin{array}{cc} \cos\theta & -\sin\theta \\ \sin\theta & \cos\theta \end{array}). \end{array}$$ (19)

We are interested in the first row of this matrix, which describes the projection onto direction *θ*. Let **Y**_*i*_ = (*Y*_*i*1_, *Y*_*i*2_)^*T*^ = **R**(**X**_*i*_ − ***μ***) for *i* = 1, 2 respectively, where ***μ*** = (*μ*_1_, *μ*_2_)^*T*^ is a shift vector mentioned in (15). Basic multivariate theory shows that

$$\begin{array}{c} {\text{Y}}_{i}∼N_{2}\{\text{R}\left(\nu_{i}-\mu \right),\;\text{R}\Sigma_{i}{\text{R}}^{T}\},\;\;\;\;\;\;\text{for}\;i=1,2\text{.} \end{array}$$ (20)

Denote **Y**_*i*_ = (*Y*_*i*1_, *Y*_*i*2_)^*T*^, *Y*_*i*1_ is the first entry of **Y**_*i*_ for *i* = 1, 2. Then, it is immediate that $Y_{i1}∼N\left(s_{i},\;\sigma_{i}^{2}\right)$, where

$$s_{1}=\left(\nu_{11}-\mu_{1}\right)\cos\theta -\left(\nu_{12}-\mu_{2}\right)\sin\theta \;\;\text{and}\;\;\sigma_{1}^{2}={\left(\text{R}\Sigma_{1}{\text{R}}^{T}\right)}_{1,1},$$ (21)

$$s_{2}=\left(\nu_{21}-\mu_{1}\right)\cos\theta -\left(\nu_{22}-\mu_{2}\right)\sin\theta \;\;\text{and}\;\;\sigma_{2}^{2}={\left(\text{R}\Sigma_{2}{\text{R}}^{T}\right)}_{1,1}.$$ (22)

The mixture distribution that we study is

$$\begin{array}{c} f_{W}\left(w_{1},w_{2}\right)=\left(1-\epsilon \right)f_{{\text{X}}_{1}}\left(w_{1},w_{2}\right)+\epsilon f_{{\text{X}}_{2}}\left(w_{1},w_{2}\right), \end{array}$$ (23)

where $f_{{\text{X}}_{i}}$ is the density of *X*_*i*_, and *ϵ* ∈ [0, 1], is typically small. Here, $f_{{\text{X}}_{1}}$ is considered to be the bulk of the distribution and $f_{{\text{X}}_{2}}$ the outlier.

#### 2.3.2 Projected distribution

Based on the bivariate setup above, the projected distribution is

$$\begin{array}{c} f_{Y}\left(y\right)=\left(1-\epsilon \right)\phi_{s_{1},\sigma_{1}^{2}}\left(y\right)+\epsilon \phi_{s_{2},\sigma_{2}^{2}}\left(y\right), \end{array}$$ (24)

where $s_{1},s_{2},\sigma_{1}^{2},\sigma_{2}^{2}$ are as above and *ϕ* is the standard normal density.

The distribution function of the projected *Y*(*θ*) is

$$\begin{array}{c} F_{Y}\left(y\right)=\left(1-\epsilon \right)\Phi_{s_{1},\sigma_{1}^{2}}\left(y\right)+\epsilon \Phi_{s_{2},\sigma_{2}^{2}}\left(y\right), \end{array}$$ (25)

where Φ is the standard normal distribution function. We require the median of the projected distribution, i.e. find

$$\begin{array}{c} y_{m}\left(\epsilon ,\theta ,s_{1},s_{2},\Sigma_{1},\Sigma_{2}\right)\;\text{such}\;\text{that}\;F_{Y}\left(y_{m}\right)=1/2. \end{array}$$ (26)

Finding an analytic exact solution for *y*_*m*_ is difficult. Hence, we will simplify the problem and assume that Σ_1_ = Σ_2_ = *I*_2_, the identity matrix. Since **R**(*θ*) is an orthogonal matrix, this means that $\sigma_{1}^{2}=\sigma_{2}^{2}=1$ and Eq (25) becomes

$$\begin{array}{c} F_{Y}\left(y\right)=\left(1-\epsilon \right)\Phi \left(y-s_{1}\right)+\epsilon \Phi \left(y-s_{2}\right). \end{array}$$ (27)

For small *ϵ*, we know that the median should be close to the median of the bulk, so the median of *F*_*Y*_ should be close to *s*_1_, the median of the first component of the mixture in Eq (27).

#### 2.3.3 Theoretical approximation of yamm on the mixture

We derive a theoretically based approximation to the empirical influence function. We proceed by using a Taylor series expansion of *F*_*Y*_(*y*) around *s*_1_, the quantity we know is close to our median:

$$\begin{array}{cc} F_{Y}\left(y\right) & \approx [1+\epsilon -\epsilon \;\mathrm{Erfc}\{\left(s_{1}-s_{2}\right)/\sqrt{2}\}]/2\\ & +{\left(2\pi \right)}^{-1/2}[1-\epsilon +\epsilon \exp\{-{\left(s_{1}-s_{2}\right)}^{2}/2\}]\left(y-s_{1}\right)\\ & +O\{{\left(y-s_{1}\right)}^{2}\}, \end{array}$$ (28)

where $\mathrm{Erfc}\left(y\right)=2\pi^{-1/2}\int_{y}^{\infty }e^{-t^{2}}\;dt.$ When *y* is close to *s*_1_, Eq (28) is approximately equal to 1/2 when *ϵ* is small, which is the behaviour we expect.

To find an approximation to the median we solve *F*_*Y*_{*y*_*m*_(*θ*)} = 1/2. Ignoring remainders, subtracting 1/2 off both sides of Eq (28) gives

$$\begin{array}{c} \frac{\epsilon }{2}[\mathrm{Erfc}\{\left(s_{1}-s_{2}\right)/\sqrt{2}\}-1]=\frac{[1-\epsilon +\epsilon \exp\{-{\left(s_{1}-s_{2}\right)}^{2}/2\}]\left(y_{m}-s_{1}\right)}{\sqrt{2\pi }}, \end{array}$$ (29)

and then

$$\begin{array}{c} y_{m}\left(\theta \right)\approx s_{1}+\frac{\epsilon \sqrt{\pi /2}[\mathrm{Erfc}\{\left(s_{1}-s_{2}\right)/\sqrt{2}\}-1]}{\left[1-\epsilon +\epsilon \exp\{-{\left(s_{1}-s_{2}\right)}^{2}/2\}\right]}. \end{array}$$ (30)

Now using

$$\begin{array}{c} \mathrm{Erfc}\left\{\left(s_{1}-s_{2}\right)/\sqrt{2}\right\}=2\Phi \left\{\left(s_{2}-s_{1}\right)/\sqrt{2}\right\}, \end{array}$$ (31)

and $\exp\left\{-{\left(s_{1}-s_{2}\right)}^{2}/2\right\}=\sqrt{2\pi }\phi \left(s_{1}-s_{2}\right)$, we can write

$$\begin{array}{c} y_{m}\left(\theta \right)\approx s_{1}+\frac{\epsilon \sqrt{\pi /2}(2\Phi \{\left(s_{2}-s_{1}\right)/\sqrt{2}\}-1)}{1-\epsilon -\sqrt{2\pi }\epsilon \phi \left(s_{2}-s_{1}\right)}. \end{array}$$ (32)

For small *ϵ* the denominator is close to 1. From Eqs (21) and (22), we can write:

$$\begin{array}{c} s_{2}-s_{1}=\left(\nu_{21}-\nu_{11}\right)\cos\theta -\left(\nu_{22}-\nu_{12}\right)\sin\theta =\delta_{1}\cos\theta -\delta_{2}\sin\theta , \end{array}$$ (33)

where *δ*_1_ = *ν*_21_ − *ν*_11_ and *δ*_2_ = *ν*_22_ − *ν*_12_. Thus

$$\begin{array}{cc} y_{m}\left(\theta \right) & \approx \{\left(\nu_{11}-\mu_{1}\right)\cos\theta -\left(\nu_{12}-\mu_{2}\right)\sin\theta \}\\ & +\frac{\epsilon \sqrt{\pi /2}[2\Phi \{\left(\delta_{1}\cos\theta -\delta_{2}\sin\theta \right)/\sqrt{2}\}-1]}{1-\epsilon -\sqrt{2\pi }\epsilon \phi \left(\delta_{1}\cos\theta -\delta_{2}\sin\theta \right)}. \end{array}$$ (34)

According to Eq (17), our job is to find the optimal $\mu^{*}={\left(\mu_{1}^{*},\mu_{2}^{*}\right)}^{T}$, which minimises

$$\begin{array}{c} M=\int_{0}^{2\pi }y_{m}^{2}\left(\theta \right)\;d\theta . \end{array}$$ (35)

The integrand involves the standard normal distribution function, which is tricky to handle analytically. Hence, we use the approximation, *ϕ*(*z*)≈(1 + cos *z*)/2*π*, for −*π* < *z* < *π*, for the standard normal density [11], which enables the following proposition.

**Proposition**. *Let **X***_1_ = (*X*_11_, *X*_12_)^*T*^ *and **X***_2_ = (*X*_21_, *X*_22_)^*T*^. *Suppose that* ${\text{X}}_{1}∼N_{2}(\nu_{1},\Sigma_{1})$ *and* ${\text{X}}_{2}∼N_{2}(\nu_{2},\Sigma_{2})$ *independently, where **ν***_1_ = (*ν*_11_, *ν*_12_)^*T*^ *and **ν***_2_ = (*ν*_21_, *ν*_22_)^*T*^, *respectively. Let the mixture, W, of **X***_1_ *and **X***_2_ *be*

$$\begin{array}{c} f_{W}\left(w_{1},w_{2}\right)=\left(1-\epsilon \right)f_{{\text{X}}_{1}}\left(w_{1},w_{2}\right)+\epsilon f_{{\text{X}}_{2}}\left(w_{1},w_{2}\right), \end{array}$$

*where ϵ* ∈ [0, 1] *is considered small*.

*An approximation of the yamm estimator*, $\mu^{*}=\left(\mu_{1}^{*},\mu_{2}^{*}\right)$, *is*

$$\begin{array}{cc} \mu_{1}^{*}= & \;\nu_{11}+\pi^{-1/2}R\epsilon (1-R^{2}/32+R^{4}/1536)\cos\alpha ,\\ \mu_{2}^{*}= & \;\nu_{12}+\pi^{-1/2}R\epsilon (1-R^{2}/32+R^{4}/1536)\sin\alpha , \end{array}$$ (36)

*where* $R^{2}=\left(\delta_{1}^{2}+\delta_{2}^{2}\right)$, *δ*_1_ = *ν*_21_ − *ν*_11_, *δ*_2_ = *ν*_22_ − *ν*_12_ *and α* = arctan(*δ*_2_/*δ*_1_). *The approximation we use is valid whenever* $|R\cos\left(\theta +\alpha \right)|<\sqrt{2}\pi$, *where θ is the projection direction when computing yamm. This inequality is true for all θ whenever* $R<\sqrt{2}\pi$.

Intuitively, the approximation in the Proposition works whenever the two cluster means are close enough together, i.e. when $R^{2}=\delta_{1}^{2}+\delta_{2}^{2}<2\pi^{2}$.

In particular, when *ν*_11_ = *ν*_21_ or *ν*_12_ = *ν*_22_ (i.e. when one of the *δ*_*i*_ = 0, *i* = 1, 2), we can form a more accurate approximation. This is because the approximation for the standard normal distribution function, *ϕ*(*z*)≈(1 + cos *z*)/2*π*, is no longer required to find the optimal $\mu^{*}={\left(\mu_{1}^{*},\mu_{2}^{*}\right)}^{T}$ minimising Eq (35). Without loss of generality, let *ν*_1_ = (*ν*_11_, *ν*_12_)^*T*^ = (0, 0)^*T*^ and *ν*_2_ = (*ν*_21_, *ν*_22_)^*T*^ = (0, *d*)^*T*^, we obtain the yamm estimator as follows

$$\begin{array}{cc} \mu_{1}^{*} & =0,\\ \mu_{2}^{*} & =2^{-1/2}\epsilon d\;e^{-\frac{d^{2}}{8}}(\text{BesselI}[0,d^{2}/8]+\text{BesselI}[1,d^{2}/8]), \end{array}$$ (37)

where BesselI[*n*, *z*] is the modified Bessel function of the first kind, sometimes denoted *I*_*n*_(*z*). For the proof of the proposition, see S2 Appendix.

#### 2.3.4 The yamm influence curve on the mixture

This section numerically computes and plots yamm for the case where *ϵ* = 0.05, ${\text{X}}_{1}∼N_{2}\left(\nu_{1},I_{2}\right)$ and ${\text{X}}_{2}∼N_{2}\left(\nu_{2},I_{2}\right)$, with ***ν***_1_ = (0, 0)^*T*^ and ***ν***_2_ = (0, *d*)^*T*^ for d∈R. We explore how yamm varies as *d* increases from 0 to 10 in steps of 0.2. If yamm is robust, then it should increase with *d*, but plateau beyond a certain point.

**Fig 2:**
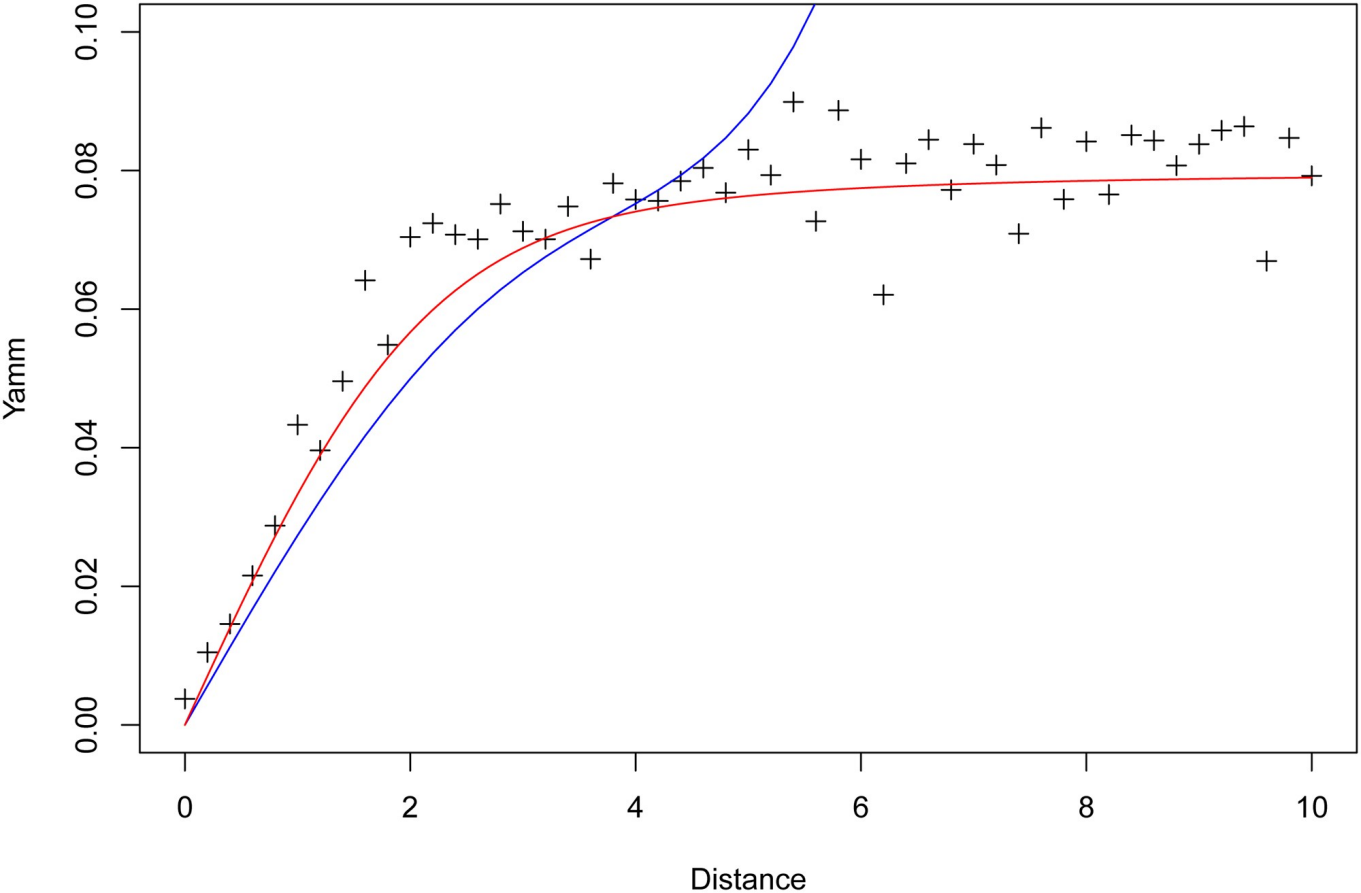
Yamm computed on simulated setup, increasing the distance between two bivariate normals. *Crosses*: numerically computed values; *Solid blue line*: approximation computed for general ***ν***_1_ and ***ν***_2_; *Solid red line*: approximation computed when ***ν***_1_ = (0, 0)^*T*^ and ***ν***_2_ = (0, *d*)^*T*^.

For each value *d* we estimate yamm as the mean over five hundred bivariate mixture realizations, with two thousand projections involved for each yamm computation, using methods described below in Section 2.4. The numerically computed crosses in Fig 2 show that, for this setup, yamm plateaus somewhere between *d* = 2 and *d* = 4.

The solid red line in Fig 2 shows our theoretical approximation of the yamm influence curve with the more specific setup, where μ* follows Eq (37). Under this approximation, the influence curve closely follows the numerically computed crosses. On the other hand, the solid blue line is the approximation of the yamm under the more general setting of Eq (36), which exhibits poor approximation after *d* > 4.5, although it performs reasonably well when the inter-cluster mean distance 0 < *d* < 4.5, and does not plateau.

This is because, in the setup, *δ*_1_ = *d*, *δ*_2_ = 0, and *d* > 4.5 implies $R^{2}=\delta_{1}^{2}=d^{2}>2\pi^{2}$. However, the specific setup approximation of yamm obviously does not work for arbitrary values of ***ν***_1_ and ***ν***_2_, whereas the general approximation gives a good theoretical idea of the yamm influence curve when the two means of the clusters are close enough together.

### 2.4 Projection median and yamm computation

#### 2.4.1 Projection median computation

A simple Monte Carlo integration [12] can be used to compute an approximation of the projection median by

$$\begin{array}{c} {\hat{M}}_{P}\left(\text{X}\right)=nJ^{-1}\sum_{j=1}^{J}\text{med}\left({\text{X}}_{{\text{a}}_{j}}\right), \end{array}$$ (38)

where *J* represents the number of projections used, and ${\left\{{\text{a}}_{j}\right\}}_{j=1}^{J}$ is a set of random, independently-drawn, unit length *n*-vectors over **X**^*n*−1^.

Calculating approximation of Eq (38) is relatively straightforward, but a large value of *J* is required to ensure accuracy. Another approach computes the projection median directly from the definition in Eq (12), using the spherical coordinates illustrated in Eq (13), where the integral can be obtained by the trapezoidal rule. For example, in the two-dimensional case, we apply the trapezoidal rule once on Eq (11). In the three-dimensional case, we have to apply the trapezoidal rule twice for the double integral, and so on. This direct approach is easy to implement when our dataset has a low dimension, but excessive work is required in not that many higher dimensions, even with, e.g. *n* = 10.

#### 2.4.2 Computing yamm

To compute an approximation to yamm, we can also use Monte Carlo integration together with an optimiser. Let J∈N be the number of projections, ${\left\{{\text{a}}_{j}\right\}}_{j=1}^{J}$ be a set of independent random unit length *n*-vectors, an estimator for *M*_**X**, *m*_(***μ***) is given by

$$\begin{array}{c} {\hat{M}}_{\text{X},m}\left(\mu \right)=J^{-1}\sum_{j=1}^{J}m_{\text{X}}{\left(\mu ,{\text{a}}_{j}\right)}^{2}. \end{array}$$ (39)

We then numerically minimise ${\hat{M}}_{\text{X},m}\left(\mu \right)$ over ***μ*** to obtain our estimated location measure, using the BFGS optimization method [13–16]. BFGS is a quasi-Newton algorithm searching for a stationary point of a function via local quadratic approximation. Parallel versions such as optimParallel exist as easy to use packages in R.

With reasonable starting values, such as the mean or other multivariate medians, yamm typically provides accurate results with a considerably smaller number of projections than used by the Monte Carlo projection median method mentioned above.

In conclusion, projection median computation via the trapezoidal rule is fast and accurate in low dimensions, but increasingly onerous in higher dimensions, as progressively more multidimensional integration is required. For higher dimensions, we prefer the Monte Carlo method and prefer yamm over the projection median as it does not require such a large number of projections, particularly if the optimiser is given a good starting solution.

Overall, approximating the projection median by the trapezoidal rule is a good choice in $R^{2}$ and $R^{3}$, and either of the other two methods can be used in higher dimensions.

## 3 Empirical performance for different medians

This section reviews the theoretical computational complexity for a variety of medians and computes some running times for real implementations of several medians computed in R. We then present some results for accuracy of estimation for these medians.

### 3.1 Computational complexity and empirical speed

For a dataset in $R^{n}$ with *k* observations, the computational complexity for the Spatial median is *O*(*nk*) [17], which is the same for the exact computation of the component-wise median. The projection median can be obtained in *O*(*k*^4/3^log^1+*ϵ*^ *k*) time in $R^{2}$ [9], and *O*(*k*^5/2+*ϵ*^) time in $R^{3}$ [10]. In $R^{n}$, with *n* > 3, Basu *et al*. showed that *O*[*k*^*n*{1−*δ*_*n*_/(*n*+1)}+*ϵ*^] time is required to compute the projection median, where *δ*_*n*_ = (4*n* − 3)^−*n*^ and *ϵ* is a fixed small constant. Several algorithms for other multivariate medians have been developed or the bivariate case. The current best algorithms for Oja’s and Liu’s medians require *O*(*k* log^3^ *k*) and *O*(*k*^4^) time, respectively [18], whereas that for the fastest bivariate Tukey median is *O*(*k* log^3^ *k*) [19]. The calculation of these three multivariate medians in higher dimensions is more complicated and approximate computation is often preferred/required.

To provide empirical assessment of the real computation speed, we apply several R software medians to simulated data. There are several R functions using different algorithms to compute one median. For example, spatial.median from the library ICSNP estimates the median with the algorithm developed by Vardi and Zhang [20], while Gmedian developed by Cardot *et al*. [21] is faster but, perhaps, less accurate. In addition, l1median [22] from library pcaPP and med from depth also provide opportunities to compute the spatial median. Hence, after some experiments, we choose the best function (evaluated in terms of speed and accuracy) for each multivariate median in $R^{2}$ and $R^{3}$ shown in Table 1. Much of the software for multivariate medians in R only works in low numbers of dimensions.

**Table 1:** R functions used for analysing different multivariate medians.

Median	Function	Package	Source
Spatial	l1median	pcaPP	[22]
CWmed	med	depth	—
Liu’s	med	depth	[23]
Tukey’s	med	depth	[24] [25]
Oja’s	ojaMedianEvo	OjaNP	[26]
Projection	PmedTrapz	Yamm	Ours

The med function can only calculate the bivariate Liu’s median, which is considerably more challenging in higher dimensions. The calculation of Tukey’s median is exact in one and two dimensions, and approximate in higher dimensions. We use the approximate Tukey’s median computation in the med function, due to numerical errors that sometimes surface when using the exact algorithm. For Oja’s median, the approximate method (evolutionary algorithm) is used instead of the exact one, as it is faster and can deal with high dimensions.

Table 2 displays mean computation times and their standard deviations across 1000 simulated datasets from the two-dimensional Laplace distribution with different numbers of observations (*k*) for each set. The results are produced by running R on a single core of an Intel i7-8750h processor with 2.20 GHz base clock using 16Gb RAM. For small *k*, Liu’s median is fastest, but its speed is not as fast as others for higher *k*. In this experiment, Oja’s median is the slowest for small *k* values, but its speed does not appear to be particularly sensitive to *k*. Hence, its speed is faster than Tukey’s median when *k* = 200. The projection median is one of the quickest when *k* is below 100, while for large *k* values, the component-wise median and the Spatial median are faster.

**Table 2:** Mean and standard deviation (s.d.) of the operation time (×10^−5^) in seconds for data in $R^{2}$.

Median		*k* = 10	*k* = 25	*k* = 50	*k* = 100	*k* = 200
Spatial	mean	27	28	30	29	28
	s.d.	44	45	57	45	45
Component-wise	mean	24	21	25	25	24
	s.d.	42	41	43	43	43
Liu’s	mean	3	6	14	49	190
	s.d.	18	24	35	66	250
Tukey’s	mean	67	210	510	970	1890
	s.d.	47	28	40	56	100
Oja’s	mean	1430	1400	1460	1410	1410
	s.d.	410	190	270	190	160
Projection	mean	7	12	18	31	60
	s.d.	26	32	39	46	49

The results in Table 2 are produced by only one possible R function for one median. However, other functions can be used. For example, the med function from the depth package can also be used to calculate the spatial median and provides accurate answers. It is extremely fast for small *k* and lower dimensions, but it becomes slower than l1median for larger *k*. Hence, we use l1median to compute the spatial median, whose performance for small *k* is also good.

### 3.2 Mean squared error for some medians

We assess the accuracy of some of the medians via empirical mean squared error. If $\hat{\text{X}}$ is an estimator in $R^{n}$ with respect to the unknown parameter $\mu \in R^{n}$, then the mean squared error is

$$\begin{array}{c} \mathrm{MSE}\left(\hat{\text{X}}\right)=n^{-1}E(||\hat{\text{X}}-{\mu ||}_{2}^{2}), \end{array}$$ (40)

where $n^{-1}\left|\right|\hat{\text{X}}-\mu {\left|\right|}_{2}^{2}$ represents the squared Euclidean distance between $\hat{\text{X}}$ and ***μ***, normalized by the vector length. Smaller $\mathrm{MSE}(\hat{\text{X}})$ values are better.

Table 3 shows MSE results based on the same simulations as used for Table 2. Not surprisingly, for this long-tailed data, all medians perform better than the sample mean. The spatial median and the projection median have smaller mean squared error, the latter performing better for small *k* values. On the other hand, Liu’s median always produces a very high mean squared error.

**Table 3:** Mean squared error (×10^−2^) for data as in Table 2.

Location Estimator	*k* = 10	*k* = 25	*k* = 50	*k* = 100	*k* = 200
Spatial	67	21	9.7	4.6	2.3
Component-wise	74	26	12.0	5.7	2.9
Liu’s	110	31	14.0	6.3	3.2
Tukey’s	73	21	10.0	4.8	2.3
Oja’s	75	22	11.0	5.6	3.2
Projection	66	21	9.8	4.7	2.3
Mean	110	39	20.0	9.9	5.0

*Conclusion*. Based on these simulations, for the R functions listed in Table 1, the spatial and projection medians always have the lowest mean squared error, but also fast running speeds. Although Liu’s median has the shortest computation time, for small *k*, it is the most inaccurate, and its computation time becomes long for large datasets. Similarly, the component-wise median is fast, even when *k* increases, but it has a large mean squared error. Hence, the spatial and projection medians are good choices when computing two-dimensional robust measures of location in this case, and the latter is preferred for small datasets. The computational results for high-dimensional simulations (*n* = 3, 5, 10) can be found in S1 Table.

### 3.3 2D projection median computation functions

The R package DurocherProjectionMedian can be downloaded from Github at https://github.com/12ramsake/DurocherProjectionMedian.

The DurocherProjectionMedian package provides functions to compute the projection median via the Monte Carlo integration method using projectionMedianMC) [27] and an exact method for two dimensions proposed by Ramsay [28] using projectionMedian2D. Tables 4 and 5 show the performance of the different functions computing the two-dimensional projection median of 1000 simulated datasets from the Laplace distribution with different *k*.

**Table 4:** Mean and standard deviation (s.d.) of the operation time (×10^−5^) in seconds for different R functions to produce the projection median.

		*k*				
R Function		10	25	50	100	200
PmedTrapz	mean	7	12	18	31	60
	s.d.	26	32	39	46	49
projectionMedian2D	mean	320	1020	3930	11640	44830
	s.d.	50	99	420	970	2690
PmedMCInt	mean	250	320	490	870	1670
	s.d.	40	39	33	50	58
projectionMedianMC	mean	930	970	1010	1130	1280
	s.d.	49	57	65	60	55

**Table 5:** Mean squared error (×10^−3^) for 1000 sets of data in $R^{2}$ generated from Laplace distribution.

	*k*				
R Function	10	25	50	100	200
PmedTrapz	656	207	98.2	47.1	23.2
projectionMedian2D	656	206	97.4	46.9	22.9
PmedMCInt	659	205	97.8	47.0	23.0
projectionMedianMC	659	205	97.6	47.0	23.0

For the Monte Carlo Integration method, when *k* is small (e.g. under 150 in $R^{2}$), the computation time of projectionMedianMC is longer than our PmedMCInt under the same number of projections in both $R^{2}$ and high dimensions, whereas both implementations have almost the same MSE.

Although the projectionMedian2D provides a slightly smaller MSE, its running time is slow. Our PmedTrapz is faster and its MSE performance is comparable to projectionMedian2D, and, hence, the former might be recommended as the best choice for $R^{2}$.

## 4 The yamm R package

Our Yamm R package provides users with functions to compute the projection median according to the different methods mentioned in section 2.4. PmedMCInt computes the projection median using the Monte Carlo approximation; PmedTrapz uses the trapezoidal rule and currently, it is only valid in two and three dimensions; yamm computes the projection median using the Monte Carlo approximation to find the shift vector ***μ*** minimising our objective function yamm.obj. The package also includes functions Plot2dMedian and Plot2dMedian to plot different multivariate medians for data in both $R^{2}$ and $R^{3}$. Most functions in our package are implemented internally using C code. This section provides some brief illustrations of the use of Yamm.

### 4.1 Yamm projection medians

The function PmedMCInt computes the projection median for any multivariate data, x, by invoking

PmedMCInt(x, nprojs = 20000)

Since this function uses Monte Carlo integration, we need to choose the number of projections *J*, which has a default value of 20000. Typically, a large *J* is required to obtain a stable answer, which means the result will not change much if recomputed under the same conditions. This function returns the projection median estimate vector.

The function PmedTrapz computes the projection median in $R^{2}$ and $R^{3}$ and is invoked by

PmedTrapz(x, no.subinterval)

PmedTrapz applies the trapezoidal rule once in $R^{2}$ and twice in $R^{3}$ on each entry of the vector $\text{med}({\text{X}}_{\text{a}})$, mentioned in section 2.1.2, and returns a vector of the projection median estimate.

The argument no.subinterval determines the number of subintervals for the trapezoidal rule. For the bivariate case the no.subinterval argument is a single number that controls the number of subdivisions for the one-dimensional integration; for the trivariate case the argument is a vector of length two that controls the number of subdivisions for the two integrals. In general, it is better to use at least 36 subintervals, which typically produces accurate results without excessive running time.

More subintervals may be appropriate for more complex datasets. For some unusual data sets it would be ideal to have a high resolution of the interval of integration in one particular region, and a relatively low resolution elsewhere, but this is beyond the scope of the current research. A small number of partitions, e.g. below 15, is not recommended for reasons of accuracy.

The yamm function is valid for data of any dimension. It uses an optimiser to provide another method to compute the projection median. The arguments are

yamm(x, nprojs = 2000, reltol = 1e-06,

xstart = l1median(x), opt.method = “BFGS”,

doabs = 0, full.results = FALSE).

The yamm function is a wrapper to minimise the the objective function yamm.obj, which uses the Monte Carlo method to approximate the squared or absolute value of the univariate median of the projection of the shifted data matrix. The nprojs argument controls the number of projections in the Monte Carlo approximation and doabs is an indicator, where 1 uses the absolute value of the univariate median and 0 forces the use of the squared value. The arguments reltol, xstart, opt.method are supplied directly to the R optimisation function optim: reltol is the tolerance for the optimiser, with default value of 10^−6^. Usually, we set a larger value (e.g. 10^−3^) to this argument, which will reduce the running time, whilst maintaining accuracy. The argument opt.method controls the selection of optimisation methods, which can be chosen from any of the four options, “BFGS”, “Nelder-Mead” [29], “CG” [30], “L-BFGS-B” [31], and “SANN” [32]. The default choice “BFGS” is relatively fast and stable in our case. See the help page of the function optim in R for further details about the different optimisation methods. The xstart argument provides the initial value for the parameters to optimise over, which plays an important role in the function yamm. A good starting point will reduce the running time and provide a more accurate result, so we use the spatial median as the default value. Other multivariate medians could be used, but they need to be fast. If full.results = TRUE, the output of this function involves a list with components obtained from the optim function, otherwise, it returns a vector containing the multivariate median estimate.

### 4.2 Some real examples

We now exhibit results for the projection medians applied to some real datasets. Our plots show different multivariate medians and the sample mean value for two simulated datasets in $R^{2}$ and $R^{3}$, respectively, allowing the methods to be compared.

#### 4.2.1 Beetle data

The famous beetle data [33] takes six measurements on 74 flea-beetles, with each belonging to one of three different species. We apply yamm and obtain the following output:

yamm(beetle, nprojs = 1000, reltol = 1e-3, doabs = 0,

full.results = TRUE)

[1] 180.19194 123.73920 49.97819 135.87913 13.62603 95.49062

$value

[1] 5.585139

$counts

function gradient

90 4

$convergence

[1] 0

$message

NULL

The yamm results show that the optimiser executed 90 calls to the objective function yamm.obj and constructed 4 gradients. The par component contains the estimate of the yamm for the beetle data. These results are not that different from the output generated by PmedMCInt, which is

PmedMCInt(beetle, nprojs = 100000)

[1] 179.54428 124.72128 50.56934 137.47363 13.23372 94.80188

For the beetle data, we chose the number of projections in yamm to be 1000, while many more projections were required (e.g. 100000) in PmedMCInt to obtain a similar and consistent result; although yamm requires optimisation. Fewer projections for the function PmedMCInt may lead inaccurate results for some components of the multivariate median. PmedTrapz is not valid in this six-dimensional case, but we will show that it has a similar output when computing projection median in two- and three-dimensions.

#### 4.2.2 Simulated Data in $R^{2}$ with three clusters

We now use the function Plot2dMedian in the package Yamm to generate and display different multivariate medians for the simulated data set clusters2d. This set contains three clusters, which are generated randomly from different independent normal distributions, and two outliers.

**Fig 3:**
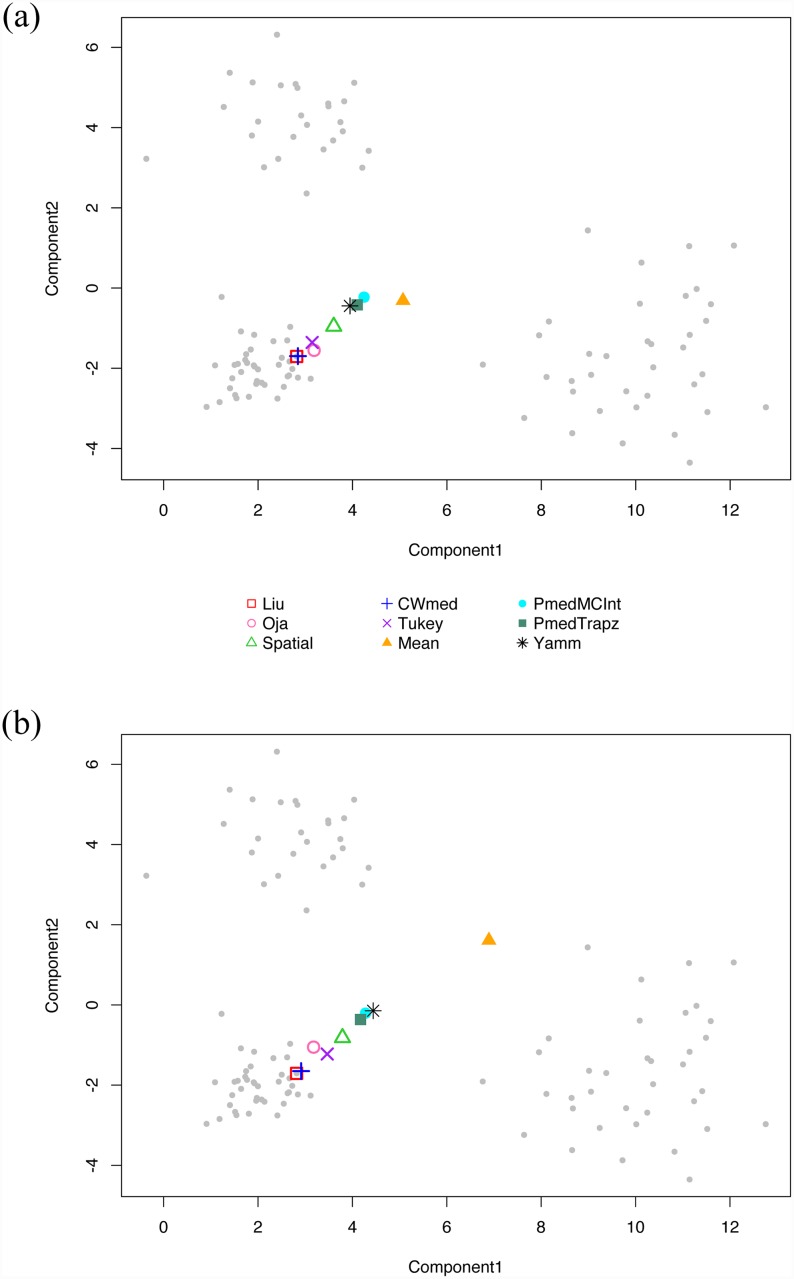
Bivariate medians and mean for three cluster two-dimensional set. *Top*: without outliers; *Bottom*: with outliers (out of plot area).

Here, we display the three different estimates of the projection median. When computing other multivariate medians, we use functions from R packages listed in section 3.1. The actual data points is plotted with grey dots. The first plot in Fig 3 is producing excluding the two outliers, whilst the second one includes them. The projection medians produced with different estimators are very close to each other, and not far from the other median estimators also. Fig 3 also shows that the multivariate medians are not particularly affect by the outliers, whilst the mean value is.

#### 4.2.3 Simulated data in $R^{3}$ with four clusters

**Fig 4:**
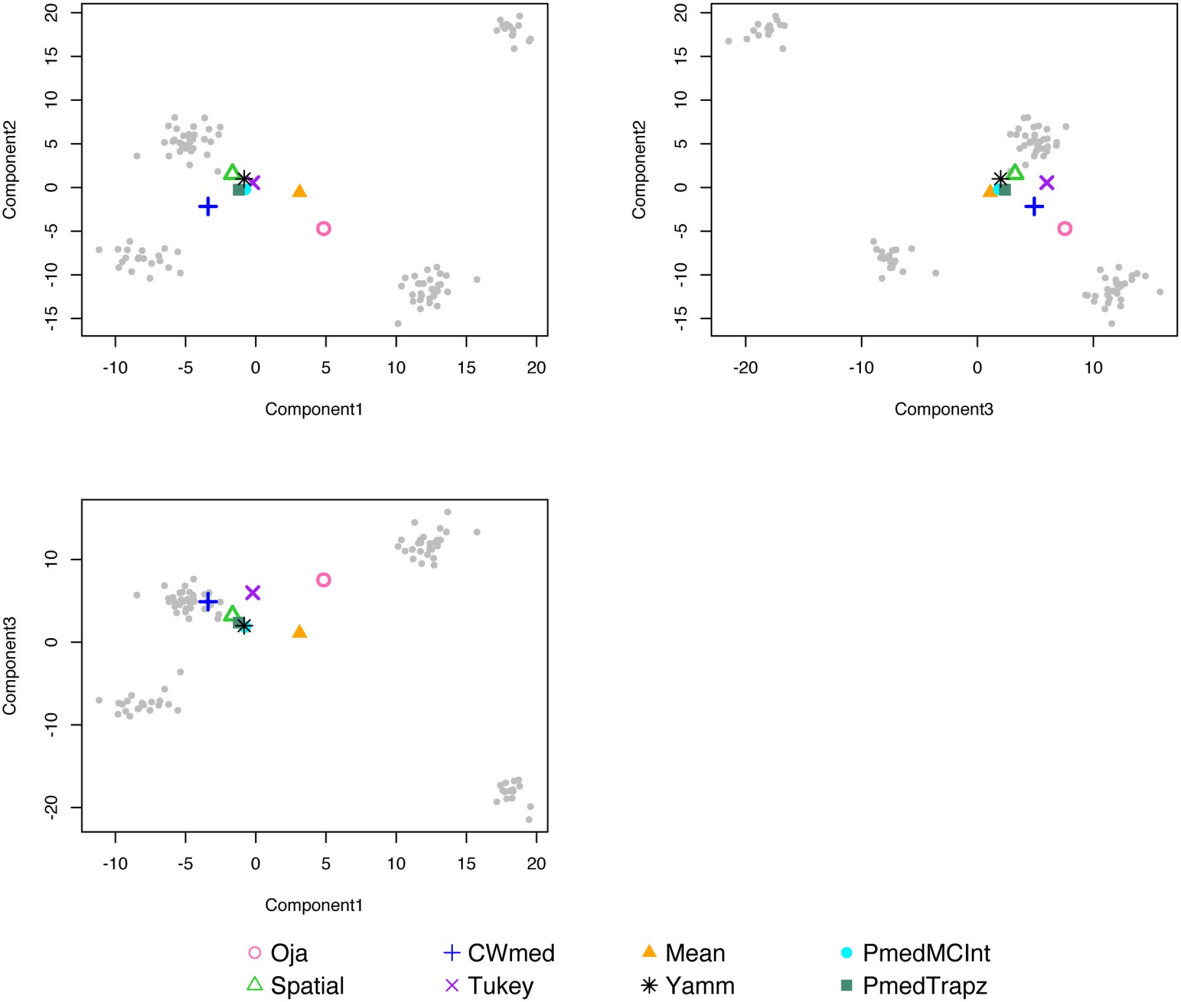
Trivariate medians & mean for four cluster three-dimensional set.

The function Plot3dMedian in Yamm plots the three-dimensional medians. The dataset clusters3d has four clusters, each generated from different independent normal distributions, as well as five outliers. Fig 4 is produced with the dataset clusters3d, whose outliers have been removed. It shows that apart from the Oja’s median, the other medians are located close to each other. Again, the three approximations of the projection median almost coincide in every component.

### 4.3 The muqie plot and some examples

As well as obtaining a robust location measure, we can use projections to provide information on the spread and configuration of the data. Obtaining true multivariate quantiles can be computationally challenging, and what we produce are not true multivariate quantiles, but they do enable us to gain useful understanding about multivariate data. The muqie (MUltivariate QuantIlE) plots are constructed as follows.

First choose a unit-length direction vector, *u*. Then project our yamm-centred multivariate data onto *u* to obtain a univariate set. The muqie point, *Q*(*α*, *u*), is merely the vector *u* rescaled to have length equal to the *α*-quantile of the univariate set. A muqie plot is the collection of all muqie points, *Q*(*α*, *u*) over all unit-length direction vectors *u*. In practice, we construct our plot by choosing a number of directions and joining the points. The basic concept, and plots, are not new, Section 2 of Fraiman and Pateiro-Lopez [34] introduces the concept based on mean-centred data and is related to ideas in [35]. Our main addition to this body of work is to (i) centre using yamm, or other robust median and (ii) presenting the muqie plots as dynamic videos of increasing *α*.

**Fig 5:**
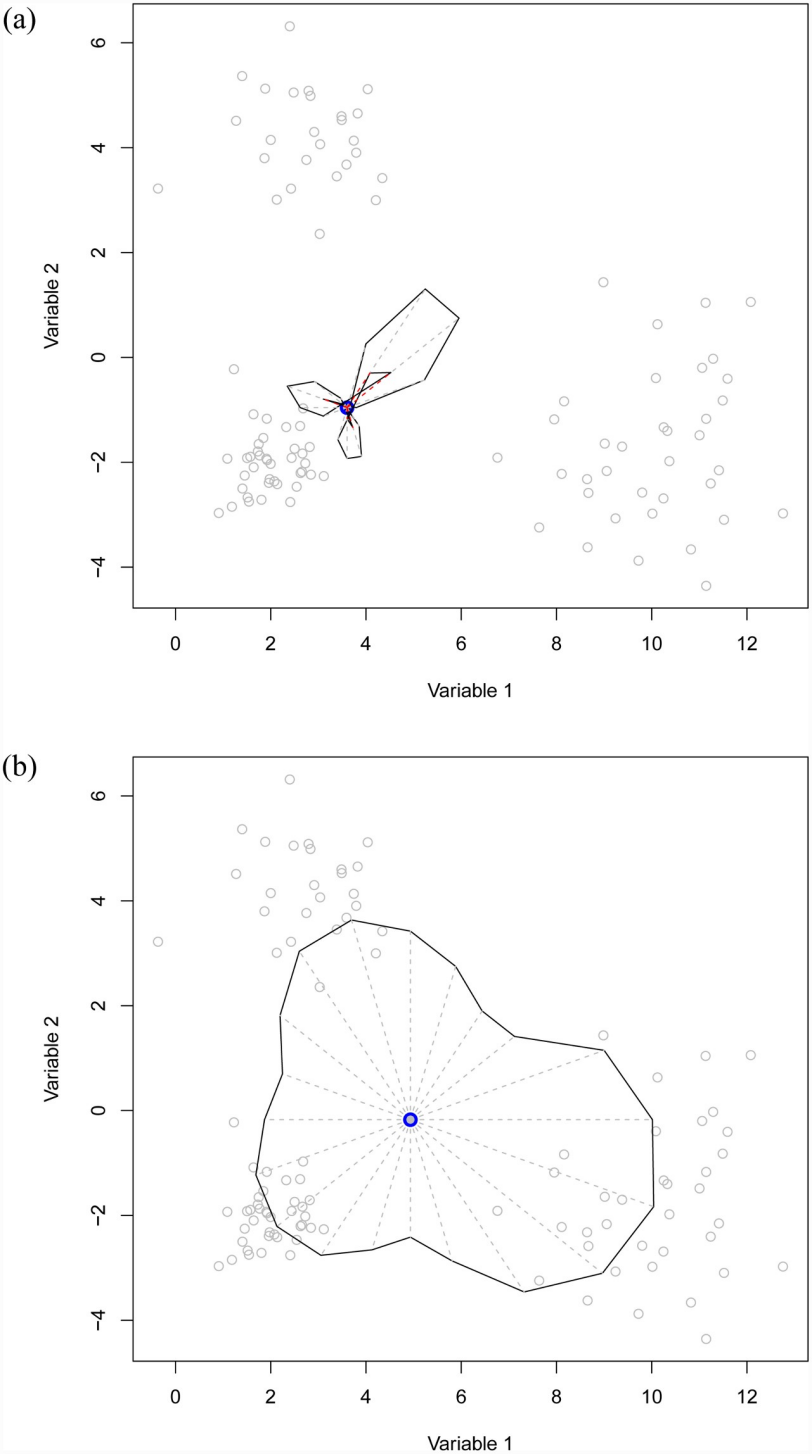
Muqie plot for the three cluster two-dimensional data set without outliers. The figures are produced for different values of pseudo-quantile *α*. The centre point (in blue) in each plot is the yamm median. Left: *α* = 0.4, Right: *α* = 0.8.

Fig 5 shows two muqie plots for *α* = 0.4 and *α* = 0.8. The latter indicates the three cluster nature. Surprisingly, this also shows up clearly in the *α* = 0.4 plot with the 0.4 quantile for, e.g. the bottom-left cluster appearing in a “north-easterly” direction and coloured red in our plot. The movie Animation shows an animated plot, which includes both the plots in Fig 5 and many of the others for increasing values of *α*.

These plots were produced by the muqie() function in the Yamm package. For the animated plot, the package includes the makeplot() function, which calls muqie() for multiple values of *α*. Then we use the CRAN package animation to produce an animated GIF using

saveGIF(makeplot(clusters2d[,-c(102,103)], nprojs = 4000),

diff.col = 3, interval = 0.1, width = 500, height = 500).

The movie beetle shows a three-dimensional Muqie plot using three variables from the beetle data. The R commands used were:

saveGIF(makeplot3D(beetle, dm = c(1,3,6)), diff.col = 3,

interval = 0.2, width = 500, height = 500)

## 5 Conclusions and discussions

We have introduced a new method, yamm, to compute the projection median, for data in $R^{n}$ with *n* ≥ 2. We have proved the theoretical equivalence of yamm and the projection median.Through theoretical and numerical investigations we demonstrate the robustness of yamm on a simple, but illuminating, bivariate setup.

Then, we illustrated three computation methods for the projection median, which can be best deployed in different situations. Approximating the projection median by the Monte Carlo method is valid in any dimensions but requires a large number of projections to ensure accuracy, while using the trapezoidal rule is computationally fast and accurate in two and three dimensions, but requires more integration on the projection vector in the higher dimensions, which becomes rapidly more complex. The yamm approximation can also compute the median in any dimensions. Its computational speed is not as quick as the other two, under the same conditions (e.g. the number of projections). However, thanks to the optimiser, a small number of the projections can be chosen to obtain an accurate median with a reasonable starting point (e.g. other multivartiate medians or mean value), which can be a distinct advantage.

Our research also documents the simulated empirical performance for different medians in terms of the computation time and the mean squared error. Using different R functions to calculate different multivariate medians, we find that the spatial median and the projection median are always accurate with relatively fast speed using the existing R functions. The performance of other multivariate medians either exhibits slow speed or large mean squared error.

Finally, we introduce our R package, Yamm, that contains our three methods to compute the projection median. We show that our methods coincide with each other in $R^{2}$ and $R^{3}$, and all multivariate medians are not affected by the outliers in the dataset, but the location of the mean value varies a lot. Currently, the function PmedTrapz in the R package is only valid in $R^{2}$ and $R^{3}$, further investment can be conducted on extending this function to higher dimensions.

The Yamm package also introduces our Muqie plots, which are capable of producing animated plots of two- and three-dimensional sets’ projected quantiles. The animated ‘growth’ of these “quantile” plots give a vivid picture of the extent, spread and configuration of data in the sets.

The Yamm package is available on the CRAN archive.

## Supporting information

S1 Appendix (PDF)

S2 Appendix (PDF)

S1 Table Simulation performance for high-dimensional medians. (PDF)

S1 File (GZ)

S1 Video (MP4)

S2 Video (MP4)
