# Supplementary material for: A new method for computing the projection median, its influence curve and techniques for the production of projected quantile plots
Source: PLoS One. 2020 May 7;15(5):e0229845. doi: 10.1371/journal.pone.0229845 (PMC7205268; doi:10.1371/journal.pone.0229845)
Supplement: S1 Appendix — (PDF) [file pone.0229845.s001.pdf]

S1 Appendix

Theorem. *For any finite multiset $\boldsymbol{X} \subseteq \mathbb{R}^n$ with $n \geq 2$, our yamm is equivalent to the projection median .*

Proof. Let $\mathbf{X} = \big(\mathbf{x}_1, \ldots, \mathbf{x}_k\big)^T \in \mathbb{R}^{k \times n}$ be a random sample of size $k \in \mathbb{N}$, $\mathbf{x}_i \in \mathbb{R}^n$. Let $\mathbf{a}$ be a $n \times 1$ projection vector of unit length, 1_k be the $k \times 1$ vector of ones and $\boldsymbol{\mu}$ a shift vector of length n. $\mathbf{y}$ is the projection of $\mathbf{X}$ onto $\mathbf{a}$ after $\mathbf{X}$ has been shifted by $\boldsymbol{\mu}$.

We now show the proof of the equivalence in 2-dimensional case, and then generalise it to the higher dimensions. Let

$$\mathbf{a} = \mathbf{a}_\theta = \Big(\cos\theta, \sin\theta\Big)^T, \tag{41}$$

$$\boldsymbol{\mu} = \Big(\mu_1, \mu_2\Big)^T, \tag{42}$$

then we have

$$\mathbf{y} = (\mathbf{X} - 1_k \boldsymbol{\mu}^T)\,\mathbf{a}_\theta \quad \text{and} \quad m_{\mathbf{X}}(\boldsymbol{\mu}, \mathbf{a}_\theta) = m(\mathbf{y}). \tag{43}$$

Hence, our objective function becomes

$$\begin{aligned}
M_{\mathbf{X},m}(\boldsymbol{\mu}) &= \int_{\theta=0}^{2\pi} m_{\mathbf{X}}(\boldsymbol{\mu}, \mathbf{a}_\theta)^2 d\theta & (44)\\
&= \int_{\theta=0}^{2\pi} \Big[m\big\{(\mathbf{X} - 1_k\boldsymbol{\mu}^T)\,\mathbf{a}_\theta\big\}\Big]^2 d\theta & (45)\\
&= \int_{\theta=0}^{2\pi} \Big\{m(\mathbf{X}\,\mathbf{a}_\theta) - \boldsymbol{\mu}^T\mathbf{a}_\theta\Big\}^2 d\theta & (46)\\
&= \int_{\theta=0}^{2\pi} m(\mathbf{X}\,\mathbf{a}_\theta)^2 - 2\,m(\mathbf{X}\,\mathbf{a}_\theta)(\boldsymbol{\mu}^T\mathbf{a}_\theta) + (\boldsymbol{\mu}^T\mathbf{a}_\theta)^2 d\theta. & (47)
\end{aligned}$$

To minimise $M_{\mathbf{X},m}(\boldsymbol{\mu})$, we want

$$
\begin{aligned}
& 0 = \frac{\partial}{\partial \mu} \int_{\theta=0}^{2\pi} m(\mathbf{X}\,\mathbf{a}_\theta)^2 - 2\,m(\mathbf{X}\,\mathbf{a}_\theta)(\boldsymbol{\mu}^T\mathbf{a}_\theta) + (\boldsymbol{\mu}^T\mathbf{a}_\theta)^2 d\theta && (48)\\
\iff\ & 0 = \int_{\theta=0}^{2\pi} \frac{\partial}{\partial \mu}\Big\{ m(\mathbf{X}\,\mathbf{a}_\theta)^2 - 2\,m(\mathbf{X}\,\mathbf{a}_\theta)(\boldsymbol{\mu}^T\mathbf{a}_\theta) + (\boldsymbol{\mu}^T\mathbf{a}_\theta)^2 \Big\} d\theta && (49)\\
\iff\ & 0 = \int_{\theta=0}^{2\pi} \frac{\partial}{\partial \mu}\Big\{ - 2\,m(\mathbf{X}\,\mathbf{a}_\theta)(\boldsymbol{\mu}^T\mathbf{a}_\theta) + (\boldsymbol{\mu}^T\mathbf{a}_\theta)^2 \Big\} d\theta && (50)\\
\iff\ & \int_{\theta=0}^{2\pi} \frac{\partial}{\partial \mu} 2\,m(\mathbf{X}\,\mathbf{a}_\theta)(\boldsymbol{\mu}^T\mathbf{a}_\theta)\mathrm{d}\theta = \int_{\theta=0}^{2\pi} \frac{\partial}{\partial \mu}(\boldsymbol{\mu}^T\mathbf{a}_\theta)^2 d\theta && (51)\\
\iff\ & \int_{\theta=0}^{2\pi} 2\mathbf{a}_\theta\, m(\mathbf{X}\,\mathbf{a}_\theta) d\theta = \int_{\theta=0}^{2\pi} 2\mathbf{a}_\theta(\boldsymbol{\mu}^T\mathbf{a}_\theta) d\theta && (52)\\
\iff\ & \int_{\theta=0}^{2\pi} \mathbf{a}_\theta\, m(\mathbf{X}\,\mathbf{a}_\theta) d\theta = \int_{\theta=0}^{2\pi} \mathbf{a}_\theta(\boldsymbol{\mu}^T\mathbf{a}_\theta) d\theta. && (53)
\end{aligned}
$$

Here, $\mathbf{a}_\theta\, m(\mathbf{X}\,\mathbf{a}_\theta)$ is the projection median of the multiset $\mathbf{X}$ on $\mathbf{a}_\theta$ in $\mathbb{R}^2$, which is defined in Eq (11) and denoted by $\mathrm{med}(\mathbf{X}_\theta)$. Also, when $\mathbf{a}_\theta = \Big(\cos\theta, \sin\theta\Big)^T$, and $\boldsymbol{\mu} = \Big(\mu_1, \mu_2\Big)^T$, we have

$$\int_{\theta=0}^{2\pi} \mathbf{a}_\theta(\boldsymbol{\mu}^T\mathbf{a}_\theta) d\theta = \pi\boldsymbol{\mu}. \tag{54}$$

Hence,

$$
\begin{aligned}
\int_{\theta=0}^{2\pi} \mathrm{med}(\mathbf{X}_\theta) d\theta &= \pi\boldsymbol{\mu} && (55)\\
\frac{1}{\pi}\int_{\theta=0}^{2\pi} \mathrm{med}(X_\theta) d\theta &= \boldsymbol{\mu}, && (56)
\end{aligned}
$$

which shows that the shift vector is the projection median in $\mathbb{R}^2$ minimising our objective function $M_{\mathbf{X},m}(\boldsymbol{\mu})$.

Our proof for higher dimensions has a similar structure. For $n > 2$, let $\boldsymbol{\mu} = \Big(\mu_1, \mu_2, \ldots, \mu_n\Big)^T$, and $\mathbf{a} = \mathbf{a}_{\theta_1,\theta_2,\ldots,\theta_{n-1}} = \Big(a_1, a_2, \ldots, a_n\Big)^T$, such that

$$
\begin{aligned}
a_1 &= \cos\theta_1\\
a_2 &= \sin\theta_1\cos\theta_2\\
a_3 &= \sin\theta_1\sin\theta_2\cos\theta_3\\
&\cdots\\
a_{n-1} &= \sin\theta_1\cdots\sin\theta_{n-2}\cos\theta_{n-1}\\
a_n &= \sin\theta_1\cdots\sin\theta_{n-2}\sin\theta_{n-1}. && (57)
\end{aligned}
$$

Taking the volume element of the hypersphere into account, we obtain the objective function as follows

$$
\begin{aligned}
M_{\mathbf{X},m}(\boldsymbol{\mu}) = \int_{\theta_{n-1}=0}^{2\pi}\int_{\theta_{n-2}=0}^{\pi}\cdots\int_{\theta_1=0}^{\pi} & m_{\mathbf{X}}(\boldsymbol{\mu},\mathbf{a})^2 \sin^{n-2}(\theta_1)\sin^{n-3}(\theta_2)\cdots\\
& \sin(\theta_{n-2})\ \ d\theta_1\cdots d\theta_{n-2}\, d\theta_{n-1}. && (58)
\end{aligned}
$$

To minimise $M_{\mathbf{X},m}(\boldsymbol{\mu})$ we require

$$\frac{\partial}{\partial \mu} M_{\mathbf{X},m}(\boldsymbol{\mu}) = 0. \tag{59}$$

After some manipulations similar to the two-dimensional case, we obtain

$$\begin{aligned}
&\int_0^{2\pi}\int_0^{\pi}\cdots\int_0^{\pi} \operatorname{med}\big(\mathbf{X}_{\theta_1,\ldots,\theta_{n-1}}\big)\sin^{n-2}(\theta_1)\cdots\sin(\theta_{n-2})d\theta_1\ldots d\theta_{n-2}\,d\theta_{n-1}\\
&=\int_0^{2\pi}\int_0^{\pi}\cdots\int_0^{\pi} \mathbf{a}(\boldsymbol{\mu}^T\mathbf{a})\sin^{n-2}(\theta_1)\cdots\sin(\theta_{n-2})d\theta_1\,\ldots\,d\theta_{n-2}\,d\theta_{n-1}.
\end{aligned} \tag{60}$$

Plugging the projection vector $\mathbf{a}$ into the right hand side of Eq (60), we have

$$\begin{aligned}
&\int_0^{2\pi}\int_0^{\pi}\cdots\int_0^{\pi} \operatorname{med}\big(\mathbf{X}_{\theta_1,\ldots,\theta_{n-1}}\big)\sin^{n-2}(\theta_1)\cdots\sin(\theta_{n-2})d\theta_1\ldots d\theta_{n-2}\,d\theta_{n-1}\\
=&n^{-1}\Big\{\int_0^{\pi}\sin^{n-2}(\theta_1)d\theta_1\cdots\int_0^{\pi}\sin(\theta_{n-2})d\theta_{n-2}\int_0^{2\pi}d\theta_{n-1}\Big\}\,\boldsymbol{\mu},
\end{aligned} \tag{61}$$

which is the definition of the projection median in higher dimensions. This means the shift vector $\boldsymbol{\mu}$ minimising our objective function $M_{\mathbf{X},m}(\boldsymbol{\mu})$ is the projection median in $\mathbb{R}^n$ with $n \geq 2$.
