# Supplementary material for: A new method for computing the projection median, its influence curve and techniques for the production of projected quantile plots
Source: PLoS One. 2020 May 7;15(5):e0229845. doi: 10.1371/journal.pone.0229845 (PMC7205268; doi:10.1371/journal.pone.0229845)
Supplement: S2 Appendix — (PDF) [file pone.0229845.s002.pdf]

S2 Appendix

Proposition. *Let* $\boldsymbol{X}_1 = (X_{11}, X_{12})^T$ *and* $\boldsymbol{X}_2 = (X_{21}, X_{22})^T$. *Suppose that* $\boldsymbol{X}_1 \sim \mathcal{N}_2\big(\boldsymbol{\nu}_1, \Sigma_1\big)$ *and* $\boldsymbol{X}_2 \sim \mathcal{N}_2\big(\boldsymbol{\nu}_2, \Sigma_2\big)$ *are random samples from bivariate normal distribution with mean vector* $\boldsymbol{\nu}_1 = (\nu_{11}, \nu_{12})^T$ *and* $\boldsymbol{\nu}_2 = (\nu_{21}, \nu_{22})^T$. *The mixture of bivariate* $\boldsymbol{X}_1$ *and* $\boldsymbol{X}_2$ *has a distribution of*

$$f_W(w_1, w_2) = (1-\epsilon) f_{\boldsymbol{X}_1}(w_1, w_2) + \epsilon f_{\boldsymbol{X}_2}(w_1, w_2),$$

where $\epsilon \in [0, 1]$ *is typically small. Let One possible approximation of the yamm,* $\boldsymbol{\mu^*} = (\mu_1^*, \mu_2^*)$, *is*

$$\begin{aligned} \mu_1^* &= \nu_{11} + \pi^{-1/2} R\epsilon \left(1 - R^2/32 + R^4/1536\right) \cos\alpha, \\ \mu_2^* &= \nu_{12} + \pi^{-1/2} R\epsilon \left(1 - R^2/32 + R^4/1536\right) \sin\alpha, \end{aligned}$$

where $R = \left\{\delta_1^2 + \delta_2^2\right\}^{1/2}$ *and* $\alpha = \arctan(\frac{\delta_2}{\delta_1})$ *with* $\delta_1 = \nu_{21} - \nu_{11}$ *and* $\delta_2 = \nu_{22} - \nu_{12}$. *The approximation is valid when* $\left|R\cos(\theta + \alpha)/\sqrt{2}\right| < \pi$, *where* θ *is the direction of the projection when computing the yamm.*

Proof. According to Section 2.3.3, we need to find the optimal $\boldsymbol{\mu^*} = (\mu_1^*, \mu_2^*)^T$ minimising

$$M = \int_0^{2\pi} y_m^2(\theta) d\theta, \tag{62}$$

where

$$\begin{aligned} y_m \approx& \left\{(\nu_{11} - \mu_1)\cos\theta - (\nu_{12} - \mu_2)\sin\theta\right\} \\ &+ \frac{\epsilon\sqrt{\pi/2}\left[2\{\Phi\{(\delta_1\cos\theta - \delta_2\sin\theta)/\sqrt{2}\} - 1\right]}{1 - \epsilon - \sqrt{2\pi}\epsilon\phi(\delta_1\cos\theta - \delta_2\sin\theta)}. \end{aligned} \tag{63}$$

We do it by splitting the integrand into three terms:

$$J_1(\theta) = \Big\{(\nu_{11}-\mu_1)\cos\theta - (\nu_{12}-\mu_2)\sin\theta\Big\}^2, \tag{64}$$

and

$$\begin{aligned} J_2(\theta) =& 2\Big\{(\nu_{11}-\mu_1)\cos\theta - (\nu_{12}-\mu_2)\sin\theta\Big\} \\ & \frac{\epsilon\sqrt{\pi/2}\,\big[2\{\Phi\{(\delta_1\cos\theta-\delta_2\sin\theta)/\sqrt{2}\}-1\big]}{1-\epsilon-\sqrt{2\pi}\epsilon\phi(\delta_1\cos\theta-\delta_2\sin\theta)}, \end{aligned} \tag{65}$$

and

$$J_3(\theta) = \frac{\pi\epsilon^2\,\big[2\{\Phi\{(\delta_1\cos\theta-\delta_2\sin\theta)/\sqrt{2}\}-1\big]^2/2}{\big\{1-\epsilon-\sqrt{2\pi}\epsilon\phi(\delta_1\cos\theta-\delta_2\sin\theta)\big\}^2}. \tag{66}$$

Now, we compute the integration term by term. The first term integration is

$$\begin{aligned} \int_0^{2\pi} J_1(\theta)\,d\theta =& \int_0^{2\pi}\Big\{(\nu_{11}-\mu_1)^2\cos^2\theta - 2(\nu_{11}-\mu_1)(\nu_{12}-\mu_2)\sin\theta\cos\theta \\ & + (\nu_{12}-\mu_2)^2\sin^2\theta\Big\}\,\mathrm{d}\theta \\ =& \pi\Big\{(\nu_{11}-\mu_1)^2 + (\nu_{12}-\mu_2)^2\Big\}. \end{aligned} \tag{67}$$

The key part of the second integrand is the "-1" part of Eq (65), which we define

$$J_{2,1}(\theta) = \frac{(\nu_{11}-\mu_1)\cos\theta - (\nu_{12}-\mu_2)\sin\theta}{1-\epsilon-\sqrt{2\pi}\epsilon\phi(\delta_1\cos\theta-\delta_2\sin\theta)}. \tag{68}$$

Now

$$J_{2,1}(\theta+\pi) = \frac{(\nu_{11}-\mu_1)\cos(\theta+\pi) - (\nu_{12}-\mu_2)\sin(\theta+\pi)}{1-\epsilon-\sqrt{2\pi}\epsilon\phi\{\delta_1\cos(\theta+\pi)-\delta_2\sin(\theta+\pi)\}} \tag{69}$$

$$= \frac{-\Big\{(\nu_{11}-\mu_1)\cos\theta - (\nu_{12}-\mu_2)\sin\theta\Big\}}{1-\epsilon-\sqrt{2\pi}\epsilon\phi\{-(\delta_1\cos\theta-\delta_2\sin\theta)\}} \tag{70}$$

$$= -J_{2,1}(\theta), \tag{71}$$

as $\cos(\theta+\pi) = -\cos\theta$, $\sin(\theta+\pi) = -\sin\theta$ and $\phi(-x) = \phi(x)$. So,

$$\int_0^{2\pi} J_{2,1}(\theta)\,d\theta = \int_0^{\pi} J_{2,1}(\theta)\,\mathrm{d}\theta + \int_\pi^{2\pi} J_{2,1}(\theta)\,d\theta \tag{72}$$

$$= \int_0^{\pi} J_{2,1}(\theta)\,d\theta + \int_0^{\pi} J_{2,1}(\theta+\pi)\,d\theta \tag{73}$$

$$= \int_0^{\pi} J_{2,1}(\theta)\,d\theta - \int_0^{\pi} J_{2,1}(\theta)\,d\theta = 0. \tag{74}$$

Hence, we only need to look at the non-"-1" part of Eq (65), and, in fact, redefine J_2 to omit that term. So, we look at

$$\begin{aligned} J_2(\theta) =& 2\{(\nu_{11}-\mu_1)\cos\theta - (\nu_{12}-\mu_2)\sin\theta \\ & \times \frac{\epsilon\sqrt{2\pi}\Phi\{(\delta_1\cos\theta-\delta_2\sin\theta)/\sqrt{2}\}}{1-\epsilon-\sqrt{2\pi}\epsilon\phi(\delta_1\cos\theta-\delta_2\sin\theta)} \end{aligned} \tag{75}$$

$$= 2\epsilon\sqrt{2\pi}\Phi\{(\delta_1\cos\theta-\delta_2\sin\theta)/\sqrt{2}\}J_{2,1}(\theta). \tag{76}$$

Note

$$J_2(\theta+\pi) = -2\epsilon\sqrt{2\pi}\Phi\{-(\delta_1\cos\theta-\delta_2\sin\theta)/\sqrt{2}\}J_{2,1}(\theta) \tag{77}$$
$$= -2\epsilon\sqrt{2\pi}\left[1-\Phi\{(\delta_1\cos\theta-\delta_2\sin\theta)/\sqrt{2}\}\right]J_{2,1}(\theta) \tag{78}$$
$$= 2\epsilon\sqrt{2\pi}\Phi\{(\delta_1\cos\theta-\delta_2\sin\theta)/\sqrt{2}\}J_{2,1}(\theta) - 2\epsilon\sqrt{2\pi}J_{2,1}(\theta) \tag{79}$$
$$= J_2(\theta) - 2\epsilon\sqrt{2\pi}J_{2,1}(\theta). \tag{80}$$

Hence

$$\int_0^{2\pi} J_2(\theta)\,\mathrm{d}\theta = \int_0^{\pi} J_2(\theta)\,d\theta + \int_{\pi}^{2\pi} J_2(\theta)\,d\theta \tag{81}$$
$$= \int_0^{\pi} J_2(\theta)\,d\theta + \int_0^{\pi} J_2(\theta+\pi)\,d\theta \tag{82}$$
$$= 2\int_0^{\pi} J_2(\theta)\,d\theta - 2\epsilon\sqrt{2\pi}\int_0^{\pi} J_{2,1}(\theta)d\theta. \tag{83}$$

Followed by Eq (83), we now consider the integral $2\int_0^{\pi} J_2(\theta)\,d\theta$ and $2\epsilon\sqrt{2\pi}\int_0^{\pi} J_{2,1}(\theta)\,d\theta$ separately.

$$2\int_0^{\pi} J_2(\theta)\,d\theta = 4\epsilon\sqrt{2\pi}\int_0^{\pi}\Phi\{(\delta_1\cos\theta-\delta_2\sin\theta)/\sqrt{2}\}J_{2,1}(\theta)d\theta. \tag{84}$$

So, consider the following integration

$$\int_0^{\pi}\Phi\{(\delta_1\cos\theta-\delta_2\sin\theta)/\sqrt{2}\}J_{2,1}(\theta)d\theta, \tag{85}$$

with

$$J_{2,1}(\theta) = \frac{(\nu_{11}-\mu_1)\cos\theta-(\nu_{12}-\mu_2)\sin\theta}{1-\epsilon\Big\{1+\sqrt{2\pi}\phi(\delta_1\cos\theta-\delta_2\sin\theta)\Big\}} \tag{86}$$
$$= \Big\{(\nu_{11}-\mu_1)\cos\theta-(\nu_{12}-\mu_2)\sin\theta\Big\}(1-x)^{-1} \tag{87}$$
$$= \Big\{(\nu_{11}-\mu_1)\cos\theta-(\nu_{12}-\mu_2)\sin\theta\Big\}\sum_{i=0}^{\infty}x^i, \tag{88}$$

by the Binomial expansion and where $x = \epsilon\{1+\sqrt{2\pi}\phi(\delta_1\cos\theta-\delta_2\sin\theta)\}$.

As we want to investigate the situation when ϵ is small, hence, for simplicity, we consider the linear terms of ϵ and ignore those terms involving higher order of ϵ. Then, we only extract the first term of $J_{2,1}(\theta)$ (i.e. $i=0$) when integrating $J_2(\theta)$. So, Eq (85) becomes

$$\int_0^{\pi}\Phi\{(\delta_1\cos\theta-\delta_2\sin\theta)/\sqrt{2}\}\Big\{(\nu_{11}-\mu_1)\cos\theta-(\nu_{12}-\mu_2)\sin\theta\Big\}d\theta. \tag{89}$$

Now, we define

$$\cos\alpha = \frac{\delta_1}{R} \quad\text{and}\quad \sin\alpha = \frac{\delta_2}{R}, \tag{90}$$
$$\cos\beta = \frac{\nu_{12}-\mu_2}{R'} \quad\text{and}\quad \sin\beta = \frac{\nu_{11}-\mu_1}{R'}, \tag{91}$$

with $R^2 = (\delta_1^2 + \delta_2^2)$, $(R')^2 = \{(\nu_{11} - \mu_1)^2 + (\nu_{12} - \mu_2)^2\}$, $\alpha = \arctan(\delta_2/\delta_1)$ and $\beta = \arctan\{(\nu_{11} - \mu_1)/(\nu_{12} - \mu_2)\}$. Using the addition formulae of trigonometric functions, then integrating by parts, Eq (89) becomes

$$\int_0^\pi -\Phi\{R\cos(\theta+\alpha)/\sqrt{2}\}\ R'\sin(\theta-\beta)d\theta \tag{92}$$

$$= \Big[\Phi\{R\cos(\theta+\alpha)/\sqrt{2}\}\ R'\cos(\theta-\beta)\Big]_0^\pi$$
$$+ \frac{RR'}{\sqrt{2}}\int_0^\pi \phi\{R\cos(\theta+\alpha)/\sqrt{2}\}\sin(\theta+\alpha)\cos(\theta-\beta)d\theta. \tag{93}$$

After some manipulations, we have

$$\Big[\Phi\{R\cos(\theta+\alpha)/\sqrt{2}\}\ R'\cos(\theta-\beta)\Big]_0^\pi = -(\nu_{12} - \mu_2) \tag{94}$$

Considering the latter part of Eq (93), we find an approximation to the standard normal density mentioned by Johnson *et al.* [11], that is

$$\phi(z) \approx \frac{1}{2\pi}(1 + \cos z), \tag{95}$$

with $-\pi < z < \pi$. Then, we expand $\cos z$ at $z = 0$ with Maclaurin series and use the first three terms for further calculation:

$$\phi(z) \approx \frac{1}{2\pi}\Big\{1 + 1 - \frac{z^2}{2!} + \frac{z^4}{4!} + O(z^6)\Big\} \tag{96}$$

$$= \frac{1}{\pi}\Big\{1 - \frac{z^2}{4} + \frac{z^4}{48} + O(z^6)\Big\}. \tag{97}$$

Let $z = R\cos(\theta+\alpha)/\sqrt{2}$, with $\big|R\cos(\theta+\alpha)/\sqrt{2}\big| < \pi$, we have 474

$$\frac{RR'}{\sqrt{2}}\int_0^\pi \phi\{R\cos(\theta+\alpha)/\sqrt{2}\}\sin(\theta+\alpha)\cos(\theta-\beta)d\theta \tag{98}$$

$$\approx \frac{RR'}{\sqrt{2}\pi}\int_0^\pi \left\{1 - \frac{R^2\cos^2(\theta+\alpha)}{8} + \frac{R^4\cos^4(\theta+\alpha)}{192}\right\}\sin(\theta+\alpha)\cos(\theta-\beta)d\theta \tag{99}$$

$$= \frac{RR'}{\sqrt{2}\pi}\left\{\int_0^\pi \sin(\theta+\alpha)\cos(\theta-\beta)d\theta - \int_0^\pi \frac{R^2\cos^2(\theta+\alpha)\sin(\theta+\alpha)\cos(\theta-\beta)}{8}d\theta + \int_0^\pi \frac{R^4\cos^4(\theta+\alpha)\sin(\theta+\alpha)\cos(\theta-\beta)}{192}d\theta\right\} \tag{100}$$

$$= \frac{RR'}{\sqrt{2}}\left\{\frac{\pi}{2}\sin(\alpha+\beta) - \frac{\pi}{64}\sin(\alpha+\beta) + \frac{\pi}{3072}\sin(\alpha+\beta)\right\} \tag{101}$$

$$= \frac{RR'}{2\sqrt{2}}\sin(\alpha+\beta)\left(1 - R^2/32 + R^4/1536\right). \tag{102}$$

Hence,

$$2\int_0^\pi J_2(\theta)d\theta \approx 4\epsilon\sqrt{2\pi}\left\{-(\nu_{12} - \mu_2) + \frac{RR'}{2\sqrt{2}}\sin(\alpha+\beta)\left(1 - \frac{R^2}{32} + \frac{R^4}{1536}\right)\right\}. \tag{103}$$

Now, consider the second part of $\int_0^{2\pi} J_2(\theta)\, d\theta$, that is

$$2\epsilon\sqrt{2\pi}\int_0^{\pi} J_{2,1}(\theta)d\theta = 2\epsilon\sqrt{2\pi}\int_0^{\pi} \Big\{(\nu_{11}-\mu_1)\cos\theta - (\nu_{12}-\mu_2)\sin\theta\Big\}\sum_{i=0}^{\infty} x^i d\theta, \quad (104)$$

where $x = \epsilon\{1 + \sqrt{2\pi}\phi(\delta_1\cos\theta - \delta_2\sin\theta)\}$.

As mentioned before, we only consider the linear terms of ϵ. Hence, Eq (104) is approximately

$$2\epsilon\sqrt{2\pi}\int_0^{\pi} \Big\{(\nu_{11}-\mu_1)\cos\theta - (\nu_{12}-\mu_2)\sin\theta\Big\}d\theta = -4\epsilon\sqrt{2\pi}(\nu_{12}-\mu_2). \quad (105)$$

Combining the results of Eq (103) and Eq (105), we have

$$\begin{aligned}\int_0^{2\pi} J_2(\theta)d\theta \approx& 4\epsilon\sqrt{2\pi}\left\{-(\nu_{12}-\mu_2) + \frac{RR'}{2\sqrt{2}}\sin(\alpha+\beta)\left(1 - \frac{R^2}{32} + \frac{R^4}{1536}\right)\right\} \\ &- \{-4\epsilon\sqrt{2\pi}(\nu_{12}-\mu_2)\} \quad (106)\\ =& 2\epsilon\sqrt{\pi}RR'\sin(\alpha+\beta)(1 - R^2/32 + R^4/1536). \quad (107)\end{aligned}$$

Finally, we wish to consider the integration of $J_3(\theta)$. When ϵ is small, the denominator of $J_3(\theta)$ is approximate to 1 and the numerator contains the quadratic terms of ϵ. As we only consider the linear terms involving ϵ, we ignore the integral $\int_0^{2\pi} J_3(\theta)\mathrm{d}\theta$. Hence, our approximation of the median becomes

$$M = \pi\Big\{(\nu_{11}-\mu_1)^2 + (\nu_{12}-\mu_2)^2\Big\} + KR'\sin(\alpha+\beta)\,\epsilon, \quad (108)$$

with $K = 2\sqrt{\pi}R(1 - R^2/32 + R^4/1536)$. Now, we wish to find μ_1 and μ_2 in terms of R (i.e. the distance between the outlier and the bulk) by partial differentiation, which will minimise the value of M.

$$\begin{aligned}\frac{M(\mu_1,\mu_2)}{\partial\mu_1} =& -2\pi(\nu_{11}-\mu_1) + K\epsilon\Big\{\frac{\partial R'}{\partial\mu_1}\sin(\alpha+\beta) + \frac{\partial\sin(\alpha+\beta)}{\partial\mu_1}R'\Big\}, \quad (109)\\ \frac{M(\mu_1,\mu_2)}{\partial\mu_2} =& -2\pi(\nu_{12}-\mu_2) + K\epsilon\Big\{\frac{\partial R'}{\partial\mu_2}\sin(\alpha+\beta) + \frac{\partial\sin(\alpha+\beta)}{\partial\mu_2}R'\Big\}. \quad (110)\end{aligned}$$

After some manipulations, we have

$$\begin{aligned}\frac{M(\mu_1,\mu_2)}{\partial\mu_1} =& -2\pi(\nu_{11}-\mu_1) - K\epsilon\Big\{(\nu_{11}-\mu_1)^2 + (\nu_{12}-\mu_2)^2\Big\}^{-1/2}\\ &\Big\{(\nu_{11}-\mu_1)\sin(\alpha+\beta) + (\nu_{12}-\mu_2)\cos(\alpha+\beta)\Big\}, \quad (111)\\ \frac{M(\mu_1,\mu_2)}{\partial\mu_2} =& -2\pi(\nu_{12}-\mu_2) - K\epsilon\Big\{(\nu_{11}-\mu_1)^2 + (\nu_{12}-\mu_2)^2\Big\}^{-1/2}\\ &\Big\{(\nu_{12}-\mu_2)\sin(\alpha+\beta) - (\nu_{11}-\mu_1)\cos(\alpha+\beta)\Big\}. \quad (112)\end{aligned}$$

Then, we set the partial derivatives of Eq (111) and Eq (112) to be 0 and solve them. Hence, we obtain the optimal values μ_1^* and μ_2^*, which minimise Eq (108), as follows:

$$\begin{aligned}\mu_1^* =& \nu_{11} + \pi^{-1/2}R\epsilon(1 - R^2/32 + R^4/1536)\cos\alpha, \quad (113)\\ \mu_2^* =& \nu_{12} + \pi^{-1/2}R\epsilon(1 - R^2/32 + R^4/1536)\sin\alpha, \quad (114)\end{aligned}$$

Finally, we plug the μ_1^* and μ_2^* into Eq (108), and obtain the minimum value of M, that is

$$M^* = R^2\epsilon^2(1 - R^2/32 + R^4/1536)\{2\sin(\alpha+\beta)+1\}. \tag{115}$$

The proof will be much simpler when $\boldsymbol{\nu}_1 = (\nu_{11}, \nu_{12})^T = (0,0)^T$ and $\boldsymbol{\nu}_2 = (\nu_{21}, \nu_{22})^T = (0,d)^T$. According to Eq (89), since $\delta_1 = 0$, $\delta_2 = d$, we have

$$2\int_0^\pi J_2(\theta)\,\mathrm{d}\theta \approx 4\epsilon\sqrt{2\pi}\int_0^\pi \Phi(-d\sin\theta/\sqrt{2})(-\mu_1\cos\theta + \mu_2\sin\theta)\mathrm{d}\theta. \tag{116}$$

Integration by part, we have

$$\begin{aligned}
2\int_0^\pi J_2(\theta)\mathrm{d}\theta \approx\ & 4\epsilon\sqrt{2\pi}\Bigg\{\Big[\Phi(-d\sin\theta/\sqrt{2})(-\mu_1\sin\theta - \mu_2\cos\theta)\Big]_0^\pi \\
& - \int_0^\pi \frac{d}{\sqrt{2}}\phi(-d\sin\theta/\sqrt{2})(\mu_1\sin\theta + \mu_2\cos\theta)\cos\theta\,\mathrm{d}\theta\Bigg\} \qquad (117)\\
=\ & 4\epsilon\sqrt{2\pi}\Bigg\{\mu_2 - \frac{d}{\sqrt{2}}\,\mu_2\int_0^\pi \phi(-d\sin\theta/\sqrt{2})\,\cos^2\theta\,\mathrm{d}\theta\Bigg\} \qquad (118)\\
=\ & 4\epsilon\sqrt{2\pi}\Bigg\{\mu_2 - \frac{d\sqrt{\pi}}{4}\,\mu_2\,e^{-\frac{d^2}{8}}\Big(\mathrm{BesselI}\big[0,\frac{d^2}{8}\big] + \mathrm{BesselI}\big[1,\frac{d^2}{8}\big]\Big)\Bigg\} \qquad (119)
\end{aligned}$$

Now, we try to compute $2\epsilon\sqrt{2\pi}\int_0^\pi J_{2,1}(\theta)\mathrm{d}\theta$ in the second part of Eq (83).

$$\begin{aligned}
2\epsilon\sqrt{2\pi}\int_0^\pi J_{2,1}(\theta)\mathrm{d}\theta &\approx -2\epsilon\sqrt{2\pi}\Big\{\int_0^\pi(-\mu_1\cos\theta + \mu_2\sin\theta)\,\mathrm{d}\theta\Big\} \qquad (120)\\
&= 4\epsilon\sqrt{2\pi}\mu_2 \qquad (121)
\end{aligned}$$

Hence,

$$\begin{aligned}
&\int_0^{2\pi} J_2(\theta)\,\mathrm{d}\theta \qquad (122)\\
=&4\epsilon\sqrt{2\pi}\Bigg\{\mu_2 - \frac{d\sqrt{\pi}}{4}\,\mu_2\,e^{-\frac{d^2}{8}}\Big(\mathrm{BesselI}\big[0,\frac{d^2}{8}\big] + \mathrm{BesselI}\big[1,\frac{d^2}{8}\big]\Big)\Bigg\} - 4\epsilon\sqrt{2\pi}\mu_2 \qquad (123)\\
=&-\sqrt{2}d\pi\epsilon\,\mu_2\,e^{-\frac{d^2}{8}}\Big(\mathrm{BesselI}\big[0,\frac{d^2}{8}\big] + \mathrm{BesselI}\big[1,\frac{d^2}{8}\big]\Big) \qquad (124)
\end{aligned}$$

When $(\nu_{11}, \nu_{12})^T = (0,0)^T$ and $(\nu_{21}, \nu_{22})^T = (0,d)^T$, we have $J_1(\theta) = (\mu_1^2 + \mu_2^2)\pi$. Ignoring the term $J_3(\theta)$, our approximation of median M is

$$\begin{aligned}
M &\approx J_1(\theta) + J_2(\theta) \qquad (125)\\
&\approx (\mu_1^2 + \mu_2^2)\pi - \sqrt{2}d\pi\epsilon\,\mu_2\,e^{-\frac{d^2}{8}}\Big(\mathrm{BesselI}\big[0,\frac{d^2}{8}\big] + \mathrm{BesselI}\big[1,\frac{d^2}{8}\big]\Big) \qquad (126)
\end{aligned}$$

To find μ_1^* and μ_2^*, we then compute

$$\begin{aligned}
\frac{M(\mu_1,\mu_2)}{\partial\mu_1} &= 2\pi\mu_1 \qquad (127)\\
\frac{M(\mu_1,\mu_2)}{\partial\mu_2} &= 2\pi\mu_2 - \sqrt{2}d\pi\epsilon\,e^{-\frac{d^2}{8}}\Big(\mathrm{BesselI}\big[0,\frac{d^2}{8}\big] + \mathrm{BesselI}\big[1,\frac{d^2}{8}\big]\Big) \qquad (128)
\end{aligned}$$

Setting $\frac{M(\mu_1,\mu_2)}{\partial\mu_1} = \frac{M(\mu_1,\mu_2)}{\partial\mu_2} = 0$, we have

$$\mu_1^* = 0 \tag{129}$$

$$\mu_2^* = \frac{\epsilon}{\sqrt{2}}\ d\ e^{-\frac{d^2}{8}} \Big(\text{BesselI}\big[0, \frac{d^2}{8}\big] + \text{BesselI}\big[1, \frac{d^2}{8}\big] \Big) \tag{130}$$

□
