# Supplementary material for: A new method for computing the projection median, its influence curve and techniques for the production of projected quantile plots
Source: PLoS One. 2020 May 7;15(5):e0229845. doi: 10.1371/journal.pone.0229845 (PMC7205268; doi:10.1371/journal.pone.0229845)
Supplement: S1 Table — (PDF) [file pone.0229845.s003.pdf]

S3 Tables Simulation Performance for High-dimensional Medians

Table 6. Mean and standard deviation (s.d.) of the operation time $(\times 10^{-5})$ **in seconds for data in** $\mathbb{R}^3$**.**

Median		$k = 10$	$k = 25$	$k = 50$	$k = 100$	$k = 200$
Spatial	mean	28	30	29	29	35
	s.d.	45	46	45	46	48
Component-wise	mean	27	28	34	31	32
	s.d.	44	45	47	46	47
Tukey's	mean	97	430	670	1170	2160
	s.d.	20	47	50	74	130
Oja's	mean	2240	2270	2220	2290	2650
	s.d.	530	630	470	640	6330
Projection	mean	24	83	200	470	1030
	s.d.	43	37	22	49	52

R functions stated in Table 1 are used.

Table 7. Mean squared error $(\times 10^{-2})$ **for** 1000 **sets of data in** $\mathbb{R}^3$ **generated from Laplace distribution.**

Location Estimator	$k = 10$	$k = 25$	$k = 50$	$k = 100$	$k = 200$
Spatial Median	57	20	9.0	4.2	2.1
Component-wise Median	70	27	12.0	5.6	2.8
Tukey's Median	66	21	9.6	4.4	2.2
Oja's Median	68	23	11.0	5.6	3.6
Projection Median	58	21	9.1	4.2	2.1
Mean	97	40	19.0	9.7	5.1

R functions stated in Table 1 are used.

Table 8. Mean and standard deviation (s.d.) of the operation time $(\times 10^{-5})$ in seconds for data in $\mathbb{R}^5$.

Median		$k = 10$	$k = 25$	$k = 50$	$k = 100$	$k = 200$
Spatial	mean	36	30	39	40	44
	s.d.	48	46	49	49	50
Component-wise	mean	36	31	36	37	40
	s.d.	48	46	48	48	49
Tukey's	mean	200	690	930	1510	2680
	s.d.	22	42	65	110	180
Oja's	mean	6520	4620	4360	4380	4390
	s.d.	4630	1700	1450	1310	1410
Projection	mean	590	670	830	1200	1930
	s.d.	47	49	48	45	47

R function `PmedMCInt` are used to produce the projection median, since `PmedTrapz` is only valid in $\mathbb{R}^2$ and $\mathbb{R}^3$. Functions for other medians are the same as the three-dimensional case.

Table 9. Mean squared error $(\times 10^{-2})$ for 1000 sets of data in $\mathbb{R}^5$ generated from Laplace distribution.

Location Estimator	$k = 10$	$k = 25$	$k = 50$	$k = 100$	$k = 200$
Spatial Median	54	19	8.2	3.9	1.9
Component-wise Median	74	28	11.7	5.6	2.7
Tukey's Median	63	20	8.7	4.1	2.0
Oja's Median	190	23	11.0	6.6	4.8
Projection Median	57	20	8.5	4.0	1.9
Mean	100	42	19	9.9	4.9

R function `PmedMCInt` are used to produce the projection median, since `PmedTrapz` is only valid in $\mathbb{R}^2$ and $\mathbb{R}^3$. Functions for other medians are the same as the three-dimensional case.

Table 10. Mean and standard deviation (s.d.) of the operation time $(\times 10^{-5})$ in seconds for data in $\mathbb{R}^{10}$.

Median		$k = 10$	$k = 25$	$k = 50$	$k = 100$	$k = 200$
Spatial	mean	53	48	60	59	66
	s.d.	50	50	49	49	48
Component-wise	mean	53	47	53	60	64
	s.d.	50	50	50	49	48
Oja's	mean	5250	17550	14780	13110	12750
	s.d.	150	9570	7550	4940	5250
Projection	mean	1150	1240	1390	1770	2540
	s.d.	77	53	62	53	140

R function `med` is not able to compute the Tukey's median when $n = 10$.

Table 11. Mean squared error $(\times 10^{-2})$ **for** 1000 **sets of data in** $\mathbb{R}^{10}$ **generated from Laplace distribution.**

Location Estimator	$k = 10$	$k = 25$	$k = 50$	$k = 100$	$k = 200$
Spatial Median	52	18	8.0	3.8	1.9
Component-wise Median	73	27	11.7	5.6	2.8
Oja's Median	990	290	23	13	12
Projection Median	55	19	8.2	3.9	1.9
Mean	100	41	20	9.8	5.0

R function `med` is not able to compute the Tukey's median when $n = 10$.
